# Synthesis and Properties of Dibenzo-Fused Naphtho[2,3-*b*:6,7-*b*′]disilole and Naphtho[2,3-*b*:6,7-*b*′]diphosphole

**DOI:** 10.3390/molecules29184313

**Published:** 2024-09-11

**Authors:** Suzuho Morishita, Chikara Hayasaka, Keiichi Noguchi, Koji Nakano

**Affiliations:** 1 Department of Applied Chemistry, Graduate School of Engineering, Tokyo University of Agriculture and Technology, 2-24-16 Naka-cho, Koganei, Tokyo 184-8588, Japan; 2 Instrumentation Analysis Center, Tokyo University of Agriculture and Technology, 2-24-16 Naka-cho, Koganei, Tokyo 184-8588, Japan; knoguchi@cc.tuat.ac.jp

**Keywords:** silole, phosphole, π-conjugated compound, UV–vis absorption property, photoluminescence

## Abstract

Silole- and phosphole-containing polycyclic aromatic compounds have attracted significant attention in the field of organic functional materials. The structure of the aromatic units has great impact on the photophysical properties of the resulting silole- and phosphole-containing polycyclic aromatic compounds. Here, dibenzo-fused naphtho[2,3-*b*:6,7-*b*′]disilole (NDS) and naphtho[2,3-*b*:6,7-*b*′]diphosphole (NDP), where a naphthalene unit is arranged between two silole and phosphole units, respectively, were designed and synthesized. The solid-state structures of them were confirmed by X-ray crystallographic analysis. The photophysical properties were evaluated by UV−vis absorption and photoluminescence spectroscopies and compared with those of their related compounds, such as dibenzo-fused silolo[3,2-*b*]silole and benzo[1,2-*b*:4,5-*b*′]disilole, ever reported. The longest wavelength absorption band of a series of silole-fused compounds was found to be red-shifted in the order benzo[1,2-*b*:4,5-*b*′]disilole < NDS < silolo[3,2-*b*]silole derivatives. For a series of phosphole-fused compounds, π-extension from phospholo[3,2-*b*]phosphole to NDP derivatives induces the lower absorption coefficient of the longest wavelength absorption band and the red-shift of the second longest wavelength absorption band. Both NDS and NDP exhibit much lower fluorescence quantum yields than their related compounds.

## 1. Introduction

**Figure 1:**
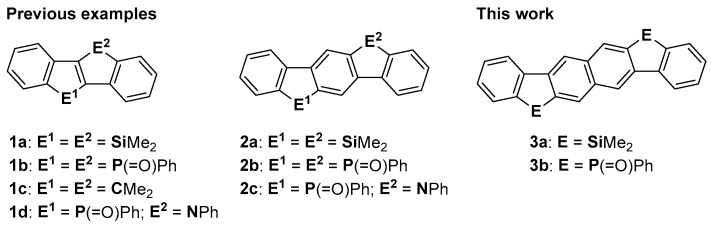
Structures of silole- and phosphole-fused polycyclic compounds **1**–**3**.

Polycyclic aromatic compounds have attracted much attention owing to their promising applications in organic electronics, such as organic light-emitting diodes [1,2], organic photovoltaics [3,4,5], and organic field-effect transistors [6,7]. The heteroatom incorporation into the π-conjugated skeletons is one of the most effective strategies in tuning the molecular structures and chemical/physical properties of polycyclic aromatic compounds. Therefore, numerous efforts have been devoted to the development of polycyclic aromatic compounds containing heteroatom(s) such as boron, nitrogen, oxygen, silicon, phosphorus, and sulfur atoms [8,9,10,11,12,13,14,15,16,17,18]. Among them, silole- and phosphole-fused compounds have been known to show intense emission [19,20,21,22,23,24,25,26,27,28]. For example, benzosilolo[3,2-*b*]benzosilole **1a** and benzophospholo[3,2-*b*]benzophosphole **1b** exhibit blue emission with a high fluorescence quantum yield (Φ) of 0.58 (λ_em_ 426 nm) and >0.98 (λ_em_ 480 nm), respectively (Figure 1) [29,30]. Their emission bands are largely red-shifted compared with that of their carbon analog **1c** (Φ 0.92; λ_em_ 322 nm). This large red-shift is attributed to the effective σ*–π* conjugation between the π* orbital of the butadiene moiety and the σ* orbital of the exocyclic Si–C and P–C bonds in the silole and phosphole units [19,31]. Such an orbital interaction decreases the energy levels of the lowest unoccupied molecular orbital (LUMO) and endows silole- and phosphole-fused compounds with intriguing properties.

The linear π-extension of polycyclic aromatic compounds has been actively investigated since the characteristics of polycyclic aromatic compounds are drastically changed by the elongation of their π-systems [9,32,33,34]. Actually, dibenzo[*d*,*d*’]benzo[1,2-*b*:4,5-*b*′]disilole and -diphosphole **2a** and **2b** have been reported as π-extended homologues of **1a** and **1b**, respectively (Figure 1) [35,36,37,38,39,40,41]. Both π-extended homologues **2a** and **2b** showed blue-shifted absorption and emission spectra in comparison with those of **1a** and **1b**. The Φ values of **2a** and **2b** are slightly lower than those of **1a** and **1b**. We have also observed the same trend for the polycyclic aromatic compounds with pyrrole and phosphole units [42]. Benzophospholo[3,2-*b*]carbazole **2c** exhibits a blue-shifted absorption edge and an emission spectrum as well as a smaller Φ value compared with benzophospholo[3,2-*b*]indole **1d**. These previous results indicate that the separation of two heterole units by a benzene unit induces blue-shifted absorption and emission bands and a lower fluorescence quantum yield.

In this context, we expected that the insertion of a naphthalene unit between two heterole units would have a significant impact on photophysical properties. As a related investigation, Imahori and co-workers have reported the thiophene-fused naphtho[2,3-*b*:6,7-*b*′]diphosphole derivative which demonstrates blue-shifted absorption and emission spectra and a smaller Φ value compared with the less π-extended thiophene-fused benzo[1,2-*b*:4,5-*b*′]diphosphole derivative [43,44]. Herein, we report the synthesis and photophysical properties of dibenzo[*d*,*d*′]naphtho[2,3-*b*:6,7-*b*′]disilole and -diphosphole **3a** and **3b**, the π-extended homologues of the silole-fused compounds **1a** and **2a**, and the phosphole-fused ones **1b** and **2b**, respectively (Figure 1). The linear π-extension from **1** to **3** allows us to understand the effect of π-extension on the photophysical properties of silole- and phosphole-fused polycyclic aromatic compounds.

## 2. Results

### 2.1. Synthesis and X-ray Crystallography

**Scheme 1:**
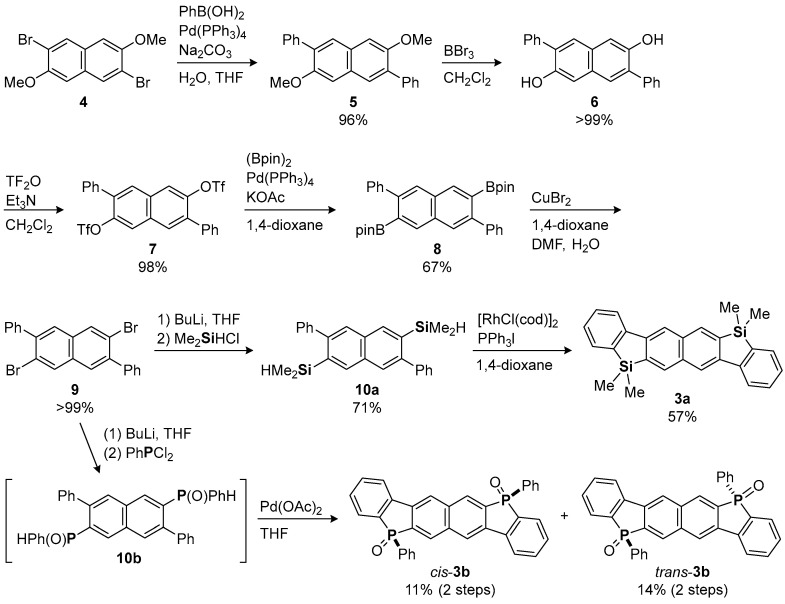
Synthesis of **3a** and **3b**.

**Figure 2:**
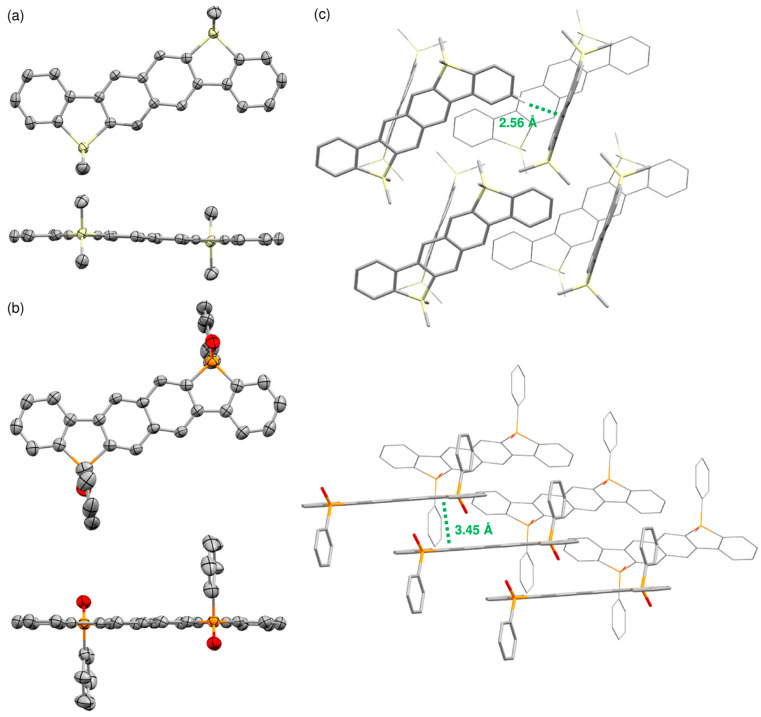
ORTEP drawing of (**a**) **3a** (upper: top view; lower: side view) and (**b**) *trans*-**3b** (upper: top view; lower: side view) [50% thermal ellipsoids. All hydrogen atoms and solvent molecules (1,4-dioxane for **3a**; chloroform for *trans*-**3b**) are omitted for clarity] and (**c**) packing structures of **3a** (upper) and *trans*-**3b** (lower).

The synthetic sequence to the target compounds is shown in Scheme 1. The silole- or phosphole-bearing fused-rings were constructed via metal-catalyzed intramolecular dehydrogenative cyclization with the corresponding bis(hydrosilane) **10a** and bis(hydrophosphine oxide) **10b** [38,40,45]. The common synthetic intermediate **9** was first synthesized. The Suzuki−Miyaura cross-coupling reaction of 2,6-dibromo-3,7-dimethoxynaphthalene (**4**) with phenylboronic acid gave 2,6-dimethoxy-3,7-diphenylnaphthalene (**5**). Then, demethylation and the following reaction with Tf_2_O gave bistriflate **7**. The (pinacolato)boron (Bpin) groups were introduced on the naphthalene core by the palladium-catalyzed borylation with bis(pinacolato)diboron. The resulting Bpin-substituted compound **8** was transformed into the common synthetic intermediate **9** using an excess amount of CuBr_2_. The obtained compound **9** was treated with BuLi, and the resulting dilithiated species was trapped by chlorodimethylsilane, affording the bis(hydrosilane) compound **10a**. Finally, the rhodium-catalyzed silylative cyclization with dehydrogenation gave NDS **3a**. The structure of **3a** was confirmed by X-ray crystallographic analysis (Figure 2). Two solvent molecules (1,4-dioxane) are included in a unit cell. The π-conjugated core was found to be slightly warped. The theoretically optimized structure of **3a** obtained at the B3LYP/6-31G+(d,p) level of theory demonstrated a completely planar structure. Therefore, there is a possibility that the packing forces in the single crystals cause the warped structure determined by X-ray analysis [44]. The π–π stacking interaction between the central π-conjugated cores is inhibited, while the C–H(aromatic)···π interaction (2.56 Å) is observed. The phosphole analog NDP **3b** was also synthesized via a transformation of compound **9** to the bis(hydrophosphine oxide) **10b** and the subsequent palladium-catalyzed dehydrogenative cyclization with the P–H and aromatic C–H bond cleavage. The final double cyclization step afforded *cis*-**3b** and *trans*-**3b** as a diastereomeric mixture. These diastereomers can be separated by silica gel column chromatography owing to the significant difference in their polarity. Their structures were verified by NMR and HRMS analyses. The identification of *trans*-**3b** by ^13^C NMR analysis was not attained because of its low solubility. Nevertheless, its structure was unambiguously identified by X-ray crystallographic analysis (Figure 2). The π-conjugated core of *trans*-**3b** is almost planar, and the one-dimensional slipped π-stacked alignment with the π–π stacking distance of 3.45 Å is observed.

### 2.2. Photophysical Properties

**Figure 3:**
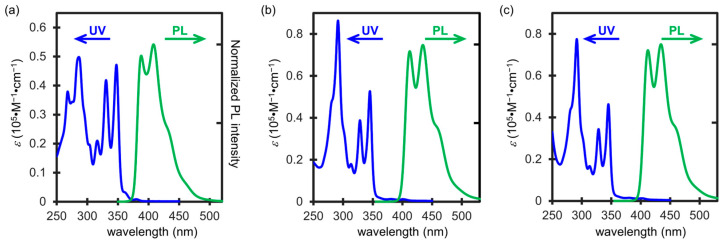
UV–vis absorption and PL spectra of (**a**) **3a**, (**b**) *cis*-**3b**, and (**c**) *trans*-**3b**.

The photophysical properties of **3a** and **3b** were evaluated by UV−vis absorption and photoluminescence (PL) spectroscopies. The obtained spectra are shown in Figure 3, and the photophysical data are summarized in Table 1, together with the data of **1a**, **1b**, **2a**, and **2b** for comparison [29,30,35,36]. The NDS derivative **3a** showed the strong vibronic π–π* transition bands with the longest absorption maximum (λ_abs_) at 347 nm in CH_2_Cl_2_ (Figure 3a). In PL measurements, NDS **3a** gave the emission maxima (λ_PL_) at 387 nm with a vibronic peak at 407 nm in CH_2_Cl_2_. Therefore, π-extension from benzene (**2a**) to naphthalene (**3a**) between two silole units was found to induce red-shifted absorption and emission maxima, while both maxima are blue-shifted compared with those of the less π-extended compound **1a**. The absolute quantum yield (Φ) of NDS **3a** is 0.08 in CH_2_Cl_2_ and 0.30 in the solid state, which are much lower than those of the less π-extended compounds **1a** and **2a**.

The *cis*/*trans* configuration of NDP has negligible impact on the absorption and emission bands (Figure 3b,c). Both NDPs *cis*-**3b** and *trans*-**3b** exhibited the strong vibronic π–π* transition bands with the absorption maximum at 345 nm (*ε* = 5.27 and 4.63 × 10^4^·M^−1^·cm^−1^ for *cis*- and *trans*-**3b**, respectively) in CHCl_3_ and much weaker absorption bands around 400 nm (*ε* = ~0.1 × 10^4^·M^−1^·cm^−1^ for *cis*- and *trans*-**3b**). According to the literature, *trans*-**1b** with a phospholo[3,2-*b*]phosphole unit exhibits the longest absorption maximum at 395 nm (*ε* = 0.69 × 10^4^·M^−1^·cm^−1^) and a stronger absorption band below 300 nm [30]. On the other hand, *trans*-**2b** shows a low-intensity absorption maximum at 383 nm (*ε* = 0.28 × 10^4^·M^−1^·cm^−1^) and a much stronger absorption band with the maximum at 330 nm (*ε*~3.0 × 10^4^·M^−1^·cm^−1^) [35]. These data indicate that π-extension from *trans*-**1b** to *trans*-**3b** induces a decrease in the absorption intensity of the longest absorption band (>350 nm) and the red-shift of the second longest wavelength absorption band (<350 nm). Both NDPs *cis*-**3b** and *trans*-**3b** exhibit an identical emission spectrum with maxima at 412 and 434 nm, which are blue-shifted compared with those of the less π-extended homologues *trans*-**1b** and *trans*-**2b**. The absolute quantum yields in the solution of NDS *cis*-**3b** and *trans*-**3b** are much lower (Φ 0.11) compared with those of **1b** and **2b**. In their solid state, *cis*-**3b** and *trans*-**3b** exhibited slightly red-shifted emission maxima. The Φ value of *trans*-**3b** (0.08) is larger than that of *cis*-**3b** (0.03), which may be attributed to the difference in their packing structure.

### 2.3. Theoretical Study on Photophysical Properties

**Figure 4:**
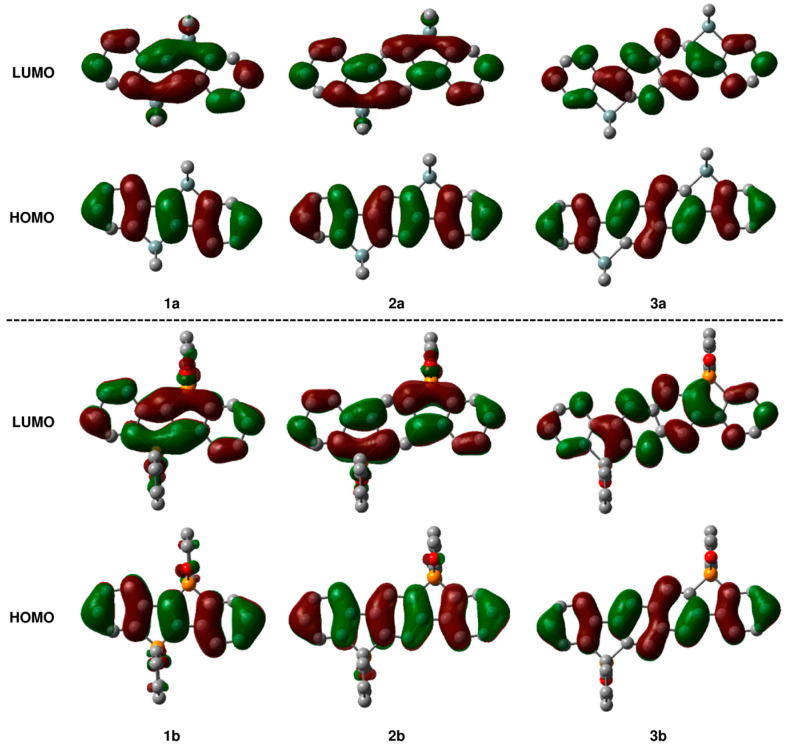
Frontier molecular orbitals of **1**–**3**.

The theoretical calculations of NDS **3a** and NDP *trans*-**3b**, and the related compounds **1a**, *trans*-**1b**, **2a**, and *trans*-**2b** were conducted by using density functional theory (DFT) and time-dependent (TD) DFT methods at the B3LYP/6-31+G(d,p) level of theory in order to understand the experimental photophysical properties. The HOMO/LUMO are described in Figure 4, and the detailed calculation results are listed in the Supporting Information. The optimized structures of all compounds were found to be highly coplanar. The HOMO and LUMO of them are distributed over the whole π-conjugated cores. No distribution on the silicon or phosphorus atom was confirmed for each HOMO of **1**–**3**. In contrast, the LUMOs of **1** and **2** possess significant distribution on silicon and phosphorus atoms, which would be attributed to the effective σ*–π* conjugation in the silole and phosphole units. It is noteworthy that the LUMOs of the π-extended compounds NDS **3a** and NDP *trans*-**3b** show no distribution on the silicon or phosphorus atom. These results indicate that the silole and phosphole units of **3a** and *trans*-**3b** have less impact on their photophysical properties compared with those of **1** and **2**.

The S_0_→S_1_ transition of NDS **3a** includes HOMO−1/LUMO, HOMO−1/LUMO, HOMO/LUMO, and HOMO/LUMO+1 transitions with an oscillator strength of 0.4782. The natural transition orbital of the S_0_→S_1_ transition shows the contribution from the silicon atoms (Figure S29). The calculated longest wavelength absorption of **3a** (358 nm, Table S9) is comparable to the experimental value (347 nm, Table 1). The red-shift of the experimental absorption maximum in the order **2a** (322 nm) < **3a** (347 nm) < **1a** (360 nm) was also well demonstrated by the TD–DFT calculations [**2a** (346 nm) < **3a** (358 nm) < **1a** (378 nm)]. The simulated absorption spectra for **1a**–**3a** fairly agree with the experimental ones (Figure S28). The calculated longest wavelength absorption band of *trans*-**3b** (373 nm) is derived from several transitions HOMO−1/LUMO, HOMO/LUMO, HOMO/LUMO+1, and HOMO−1/LUMO+1 with a small oscillator strength (*f* = 0.0244), while the oscillator strengths of the calculated longest wavelength absorption band of *trans*-**1b** (*f* = 0. 2038 at 416 nm) and *trans*-**2b** (*f* = 0.2284 at 370 nm) are much larger. The calculated second longest wavelength absorption band of *trans*-**3b** (352 nm, *f* = 0.8881) is close to that of the experimental value (345 nm) and red-shifted compared with those of *trans*-**1b** (317 nm) and *trans*-**2b** (321 nm). Accordingly, these calculation results well demonstrate the experimental results that π-extension from *trans*-**1b** to *trans*-**3b** induces a lower absorption coefficient of the longest wavelength absorption band and the red-shift of the second longest wavelength absorption band.

## 3. Materials and Methods

### 3.1. General Procedures

All manipulations involving air- and/or moisture-sensitive compounds were carried out with the standard Schlenk technique under argon. Analytical thin-layer chromatography was performed on a glass plate coated with 0.25 mm 230–400 mesh silica gel containing a fluorescent indicator. Column chromatography was performed by using silica gel (spherical neutral, particle size: 63–210 μm). Most of the reagents were purchased from commercial suppliers, such as Sigma-Aldrich Co. LLC. (St. Louis, MO, USA), Tokyo Chemical Industry Co., Ltd. (Tokyo, Japan), and Kanto Chemical Co., Inc. (Tokyo, Japan), and used without further purification unless otherwise specified. Commercially available anhydrous solvents were used for air- and/or moisture-sensitive reactions. In addition, 2,6-Dibromo-3,7-dimethoxynaphthalene (**4**) was prepared according to the literature [46].

NMR spectra were recorded in CDCl_3_ on a JEOL-ECA500 spectrometer (^1^H 500 MHz; ^13^C 126 MHz; ^19^F 471 MHz, ^31^P 202 MHz), a JEOL-ECX400 spectrometer (^1^H 400 MHz; ^13^C 101 MHz), or a JEOL-ECX300spectrometer (^1^H 300 MHz) (JEOL Ltd., Tokyo, Japan). Chemical shifts are reported in ppm relative to the internal standard signal (0 ppm for Me_4_Si in CDCl_3_) for ^1^H and the deuterated solvent signal (77.16 ppm for CDCl_3_; 29.84 ppm for acetone-d_6_) for ^13^C. Data are presented as follows: chemical shift, multiplicity (s = singlet, d = doublet, dd = doublet of doublets, t = triplet, td = triplet of doublets, m = multiplet and/or multiple resonances), coupling constant in hertz (Hz), and signal area integration in natural numbers. Melting points were determined on an SRS OptiMelt melting point apparatus (Stanford Research Systems, Sunnyvale, CA, USA). High-resolution mass spectra are taken with a Bruker Daltonics micrOTOF-QII mass spectrometer (Bruker Corporation, Billerica, MA, USA) by the atmospheric pressure chemical ionization-time-of-flight (APCI–TOF) method. UV–vis absorption spectra were recorded on a JASCO V-650 spectrophotometer (JASCO Corporation, Tokyo, Japan). Photoluminescence spectra were recorded on a JASCO FP-6500 spectrofluorometer (JASCO Corporation, Tokyo, Japan). Absolute quantum yields were determined by an absolute quantum yield measurement system with a JASCO ILF–533 integrating sphere (JASCO Corporation, Tokyo, Japan).

### 3.2. Synthesis

2,6-Dimethoxy-3,7-diphenylnaphtalene (**5**)

A mixture of compound **4** (2.1 g, 6.2 mmol), Pd(PPh_3_)_4_ (0.72 g, 0.62 mmol), and THF (62 mL) in a 300 mL three-necked flask was degassed by three freeze–thaw–pump cycles. Degassed aqueous Na_2_CO_3_ (62 mL, 2.0 M) and phenylboronic acid (1.7 g, 14 mmol) were added to the mixture under argon. The resulting mixture was further degassed by three freeze–thaw–pump cycles and stirred at 70 °C for 19 h under argon. The reaction mixture was cooled to room temperature and concentrated under reduced pressure to remove THF. Water was added to the residue, and the resulting suspension was extracted with CHCl_3_ (50 mL × 1 and 30 mL × 2). The combined organic layers were dried over Na_2_SO_4_, filtered, and concentrated under reduced pressure. The resulting crude residue was purified by silica gel column chromatography (CH_2_Cl_2_ as an eluent) to give compound **5** as a colorless solid (2.0 g, 96% yield): mp 245–247 °C; ^1^H NMR (400 MHz, CDCl_3_) δ 7.70 (s, 2H), 7.64–7.61 (m, 4H), 7.47–7.43 (m, 4H), 7.40–7.36 (m, 2H), 7.21 (s, 2H), 3.90 (s, 6H); ^13^C NMR (101 MHz, CDCl_3_) δ 154.1, 138.6, 132.8, 129.9, 129.2, 128.7, 128.1, 127.3, 106.1, 55.7; HRMS–APCI^+^ (*m*/*z*) calcd for C_24_H_21_O_2_^+^ ([M + H]^+^) 341.1537, found 341.1531.

2,6-Dihydroxy-3,7-diphenylnaphtalene (**6**)

To a mixture of compound **5** (1.7 g, 5.0 mmol) and CH_2_Cl_2_ (50 mL) in a 300 mL three-necked flask was added BBr_3_ (5.33 mL of 1.0 M solution in CH_2_Cl_2_, 5.33 mmol) dropwise at 0 °C under argon. The resulting mixture was allowed to warm to room temperature and stirred for 20 h. After the reaction was quenched with H_2_O (100 mL), the resulting mixture was extracted with CH_2_Cl_2_ (50 mL × 2) and ethyl acetate (40 mL × 2). The combined organic layers were washed with saturated aqueous NaHCO_3_ (50 mL), dried over Na_2_SO_4_, filtered, and concentrated under reduced pressure to give compound **6** as a yellowish brown solid (1.6 g, >99% yield): mp 232–237 °C; ^1^H NMR (300 MHz, CDCl_3_) *δ* 7.63 (s, 2H), 7.60–7.51 (m, 8H), 7.49–7.43 (m, 2H), 7.30 (s, 2H), 5.13 (s, 2H); ^13^C NMR (126 MHz, acetone-*d*_6_) *δ* 151.8, 139.8, 132.5, 130.4, 130.3, 128.8, 128.4, 127.7, 110.8; HRMS–APCI^+^ (*m/z*) calcd for C_22_H_17_O_2_^+^ ([M + H]^+^) 313.1224, found 313.1222.

3,7-Diphenylnaphtalene-2,6-diyl bis(trifluoromethanesulfonate) (**7**)

To a mixture of compound **6** (125 mg, 0.40 mmol), triethylamine (333 µL, 2.4 mmol), and CH_2_Cl_2_ (4 mL) in a 20 mL Schlenk tube was added trifluoromethanesulfonic anhydride (144 µL, 0.88 mmol) dropwise at 0 °C under argon. The resulting mixture was allowed to warm to room temperature and stirred for 22 h. Then, 1 M aqueous HCl (5 mL) was added to the reaction mixture, and the resulting mixture was extracted with CH_2_Cl_2_ (10 mL × 3). The combined organic layers were dried over Na_2_SO_4_, filtered, and concentrated under reduced pressure. Water (40 mL) was added to the resulting residue, and the resulting suspension was extracted with CH_2_Cl_2_ (10 mL × 3). The combined organic layers were dried over Na_2_SO_4_, filtered, and concentrated under reduced pressure to give compound **7** as a colorless solid (225 mg, 98% yield): mp 182–185 °C; ^1^H NMR (400 MHz, CDCl_3_) *δ* 8.01 (s, 2H), 7.93 (s, 2H), 7.57–7.48 (m, 10H); ^13^C NMR (101 MHz, CDCl_3_) *δ* 146.3, 135.6, 135.1, 131.8, 131.2, 129.7, 128.9, 128.8, 120.4, 118.5 (q, *J* = 321 Hz); ^19^F NMR (471 MHz, CDCl_3_) *δ* −73.6; HRMS–APCI^+^ (*m/z*) calcd for C_24_H_14_F_6_O_6_S_2_^+^ ([M]^+^) 576.0131, found 576.0147.

2,2’-(3,7-Diphenyllnaphthalene-2,6-diyl)bis(4,4,5,5-tetramethyl-1,3,2-dioxaborolane) (**8**)

A mixture of **f** (174 mg, 0.30 mmol), KOAc (174 mg, 1.8 mmol), Pd(PPh_3_)_4_ (35 mg, 30 μmmol), bis(pinacolato)diborane (231 mg, 0.91 mmol), and 1,4-dioxane (3.5 mL) in a 20 mL Schlenk tube was degassed by three freeze–thaw–pump cycles. The resulting mixture was stirred at 80 °C for 45 h under argon and concentrated under reduced pressure. Water (30 mL) was added to the residue, and the resulting suspension was extracted with CHCl_3_ (30 mL × 3). The combined organic layers were dried over Na_2_SO_4_, filtered, and concentrated under reduced pressure. The resulting crude residue was purified by silica gel column chromatography [hexane/CH_2_Cl_2_ (1/1) as an eluent, *R*_f_ = 0.31] to give **g** as a colorless solid (107 mg, 67% yield): mp 310–317 °C; ^1^H NMR (400 MHz, CDCl_3_) *δ* 8.26 (s, 2H), 7.87 (s, 2H), 7.52–7.50 (m, 4H), 7.42–7.38 (m, 4H), 7.36–7.32 (m, 2H), 1.23 (s, 24H); ^13^C NMR (101 MHz, CDCl_3_) *δ* 143.7 143.2, 135.8, 132.5, 129.4, 127.9, 127.6, 126.9, 83.9, 24.7; HRMS–APCI^+^ (*m/z*) calcd for C_34_H_38_B_2_O_4_^+^ ([M]^+^) 532.2951, found 532.2980.

2,6-Dibromo-3,7-diphenylnaphtalene (**9**)

A mixture of compound **8** (108 mg, 0.20 mmol), CuBr_2_ (270 mg, 1.2 mmol), DMF (1.8 mL), H_2_O (0.90 mL), and 1,4-dioxane (1.8 mL) in a 20 mL Schlenk tube was stirred at 95 °C for 2 h. CuBr_2_ (146 mg, 0.65 mmol) was added at 95 °C, and the resulting mixture was stirred at the same temperature for 2 h. After adding CuBr_2_ (141 mg, 0.63 mmol) at 95 °C again, the resulting mixture was stirred at the same temperature for 18 h. The reaction mixture was cooled to room temperature and concentrated under reduced pressure. Then, 1M aqueous HCl (10 mL) was added to the residue, and the resulting suspension was extracted with CHCl_3_ (20 mL × 1 and 10 mL × 3). The combined organic layers were dried over Na_2_SO_4_, filtered, and concentrated under reduced pressure to give compound **9** as a pale pink solid (88 mg, >99% yield): mp 213–216 °C; ^1^H NMR (400 MHz, CDCl_3_) *δ* 8.15 (s, 2H), 7.73 (s, 2H), 7.50–7.42 (m, 10H); ^13^C NMR (126 MHz, CDCl_3_) *δ* 141.3, 140.7, 132.5, 131.7, 129.8, 128.7, 128.2, 128.0, 121.8; HRMS–APCI^+^ (*m/z*) calcd for C_22_H_14_Br_2_^+^ ([M + H]^+^) 435.9457, found 435.9460.

2,6-Dibromo-3,7-diphenylnaphtalene (**10a**)

To a mixture of compound **9** (250 mg, 0.57 mmol) and THF (5 mL) in a 30 mL Schlenk tube was added butyllithium (0.67 mL of 2.6 M solution in hexane, 1.7 mmol) dropwise at −78 °C under argon. The resulting mixture was stirred at the same temperature for 1 h. After ClMe_2_SiH (0.19 mL, 1.7 mmol) was added to the reaction mixture, the resulting mixture was warmed to room temperature over a period of 4 h and stirred for 16 h. Saturated aqueous NH_4_Cl was added to the reaction mixture, and the resulting mixture was extracted with CH_2_Cl_2_ (20 mL × 3). The combined organic layers were dried over Na_2_SO_4_, filtered, and concentrated under reduced pressure. The resulting crude residue was purified by silica gel column chromatography (hexane as an eluent, *R*_f_ = 0.62) to give compound **10a** as a colorless solid (161 mg, 71% yield): mp 158–162 °C; ^1^H NMR (400 MHz, CDCl_3_) *δ* 8.11 (s, 2H), 7.78 (s, 2H), 7.45–7.40 (m, 10H), 4.47 (sep, *J* = 3.7 Hz, 2H), 0.11 (d, *J* = 3.7 Hz, 12H); ^13^C NMR (101 MHz, CDCl_3_) *δ* 145.8, 143.7, 136.1, 136.0, 132.0, 129.6, 128.1, 127.5, 127.3, −2.8; HRMS–APCI^+^ (*m/z*) calcd for C_26_H_28_Si_2_^+^ ([M]^+^) 396.1724, found 396.1723.

5,5,12,12-Tetramethyldibenzo[*d*,*d*’]naphtho[2,3-*b*:6,7-*b*′]disilole (**3a**)

A mixture of [RhCl(cod)]_2_ (6.4 mg, 13 μmol), PPh_3_ (20 mg, 76 μmol), and 1,4-dioxane (1 mL) in a 20 mL Schlenk tube was stirred at room temperature for 15 min under argon. After compound **10a** (171 mg, 0.43 mmol) was added to the reaction mixture, the resulting mixture was degassed by three freeze–thaw–pump cycles, stirred at 90 °C for 67 h under argon, and concentrated under reduced pressure. The resulting crude residue was purified by silica gel column chromatography (hexane as an eluent, *R*_f_ = 0.25) to give compound **3a** as a colorless solid (96 mg, 57% yield): mp 359–364 °C; ^1^H NMR (500 MHz, CDCl_3_) *δ* 8.22 (s, 2H), 8.15 (s, 2H), 8.01 (d, *J* = 8.0 Hz, 2H), 7.69 (d, *J* = 6.9 Hz, 2H), 7.50 (td, *J* = 7.4, 1.1 Hz, 2H), 7.33 (t, *J* = 6.6 Hz, 2H), 0.50 (s, 12H); ^13^C NMR (126 MHz, CDCl_3_) *δ* 147.9, 144.5, 139.7, 138.3, 134.7, 134.0, 133.0, 130.5, 127.7, 121.4, 119.2, −2.5; HRMS–APCI^+^ (*m/z*) calcd for C_26_H_25_Si_2_^+^ ([M + H]^+^) 393.1489, found 393.1489.

5,12-Diphenyldibenzo[*d*,*d*’]naphtho[2,3-*b*:6,7-*b*′]diphosphole 5,12-dioxide (**3b**)

To a mixture of compound **9** (221 mg, 0.50 mmol) and THF (10 mL) in a 50 mL Schlenk tube was added butyllithium (0.57 mL of 2.65 M solution in hexane, 1.5 mmol) dropwise at −78 °C under argon. After being stirred at the same temperature for 2 h, PhPCl_2_ (205 µL, 1.51 mmol) was added to the reaction mixture. The resulting mixture was allowed to warm to room temperature and stirred for 21 h. H_2_O (20 mL) was added to the reaction mixture, and the resulting mixture was extracted with CH_2_Cl_2_ (40 mL × 1, 20 mL × 2). The organic layers were dried over Na_2_SO_4_, filtered, and concentrated under reduced pressure. The resulting residue was purified by silica gel column chromatography [ethyl acetate/methanol/triethylamine (100/1/1) as an eluent] to give compound **10b** (127 mg) as a diastereomeric mixture with inseparable byproducts, which was used for the next step without further purification: ^31^P NMR (202 MHz, CDCl_3_) *δ* 19.1, 19.0; HRMS–APCI^+^ (*m/z*) calcd for C_34_H_27_O_2_P_2_^+^ ([M + H]^+^) 529.1481, found 529.1485.

A mixture of compound **10b** (127 mg), Pd(OAc)_2_ (6.0 mg, 0.027 mmol), and THF (1.0 mL) in a 50 mL Schlenk tube was degassed by three freeze–thaw–pump cycles and stirred at 65 °C for 48 h under argon. The reaction mixture was concentrated under reduced pressure, and the resulting residue was purified by silica gel column chromatography [CHCl_3_/acetone/methanol (100/20/1) as an eluent, *R*_f_ = 0.33 (*trans*-isomer), 0.13 (*cis*-isomer)] to give compound *trans***-3b** as a yellow solid (36 mg, 14% yield, 2 steps) and *cis***-3b** as a yellow solid (29 mg, 11% yield, 2 steps): *trans***-3b** ^1^H NMR (500 MHz, CDCl_3_) *δ* 8.30 (d, *J* = 10.9 Hz, 2H), 8.21 (d, *J* = 2.9 Hz, 2H), 7.98 (dd, *J* = 7.5, 2.9 Hz, 2H), 7.78 (dd, *J* = 9.7, 7.5 Hz, 2H), 7.72–7.65 (m, 6H), 7.53 (td, *J* = 7.2, 1.2 Hz, 2H), 7.48–7.42 (m, 6H); ^31^P NMR (202 MHz, CDCl_3_) *δ* 33.1; HRMS–APCI^+^ (*m/z*) calcd for C_34_H_23_O_2_P_2_^+^ ([M + H]^+^) 525.1168, found 525.1167; *cis***-3b** ^1^H NMR (500 MHz, CDCl_3_) δ 8.26 (d, *J* = 10.9 Hz, 2H), 8.16 (d, *J* = 2.9 Hz, 2H), 7.94 (dd, *J* = 7.5, 2.9 Hz, 2H), 7.74 (dd, *J* = 9.7, 7.5 Hz, 2H), 7.68–7.64 (m, 4H), 7.60 (t, *J* = 7.4 Hz, 2H), 7.51–7.48 (m, 2H), 7.38 (td, *J* = 7.6, 3.2 Hz, 6H); ^13^C NMR (126 MHz, CDCl_3_) *δ* 141.5 (d, *J*_C,P_ = 20.4 Hz), 138.3 (d, *J*_C,P_ = 22.8 Hz), 135.9 (d, *J*_C,P_ = 13.2 Hz), 134.0, 133.8 (d, *J*_C,P_ = 105.6 Hz), 132.9 (d, *J*_C,P_ = 108.0 Hz), 132.4, 131.9 (d, *J*_C,P_ = 9.6 Hz), 131.2 (d, *J*_C,P_ = 10.8 Hz), 131.0 (d, *J*_C,P_ = 105.6 Hz), 129.91 (d, *J*_C,P_ = 8.4 Hz), 129.86 (d, *J*_C,P_ = 10.8 Hz), 128.9 (d, *J*_C,P_ = 12.0 Hz), 122.2 (d, *J*_C,P_ = 9.6 Hz), 121.3 (d, *J*_C,P_ = 9.6 Hz); ^31^P NMR (202 MHz, CDCl_3_) *δ* 32.8; HRMS–APCI^+^ (*m/z*) calcd for C_34_H_23_O_2_P_2_^+^ ([M + H]^+^) 525.1168, found 525.1165.

### 3.3. X-ray Crystallography

For X-ray crystallographic analyses, suitable single crystals were selected under ambient conditions, mounted using a nylon loop filled with paraffin oil, and transferred to the goniometer of a RIGAKU R-AXIS RAPID diffractometer with graphite-monochromated Cu−Kα irradiation (λ = 1.54187 Å) (Rigaku, Tokyo, Japan). The structures were solved by a direct method (SIR 2008 [47]) and refined by full-matrix least-squares techniques against *F*2 (SHELXL-2014 [48,49]). The intensities were corrected for Lorentz and polarization effects. All non-hydrogen atoms were refined anisotropically. Hydrogen atoms were placed using AFIX instructions.

Crystal data for **3a**: formula: C_26_H_24_Si_2_·C_4_H_8_O_2_ (*M* = 480.73 g/mol): monoclinic, space group *P*2_1_/*c* (No. 14), *a* = 8.00343(18) Å, *b* = 12.0224(2) Å, *c* = 14.4880(3) Å, *β* = 106.8188(12)°, *V* = 1334.42(5) Å^3^, *Z* = 2, *T* = 203(2) K, *μ*(CuKα) = 1.54187 mm^−1^, *D*_calc_ = 1.196 g/cm^3^, 23203 reflections measured (4.868° ≤ *θ* ≤ 68.201°), 2453 unique (*R*_int_ = 0.0245; *R*_sigma_ = 0.0114) which were used in all calculations. The final *R*_1_ was 0.0481 (*I* > 2σ(*I*)) and w*R*_2_ was 0.1240 (all data).

Crystal data for *trans*-**3b**: formula: C_17_H_11_OP·CHCl_3_ (*M* = 381.60 g/mol): monoclinic, space group *P*2_1_/*c* (No. 15), *a* = 7.75254(14) Å, *b* = 18.8395(3) Å, *c* = 12.3790(2) Å, *β* = 101.6418(7)°, *V* = 1770.81(6) Å^3^, *Z* = 4, *T* = 203(2) K, *μ*(CuKα) = 1.54187 mm^−1^, *D*_calc_ = 1.431 g/cm^3^, 32218 reflections measured (4.337° ≤ *θ* ≤ 68.224°), 3227 unique (*R*_int_ = 0.0294; *R*_sigma_ = 0.0133) which were used in all calculations. The final *R*_1_ was 0.0471 (*I* > 2σ(*I*)) and w*R*_2_ was 0.1389 (all data).

CCDC 2376224 (**3a**) and 2376225 (*trans*-**3b**) contain the supplementary crystallographic data for this paper. The data can be obtained free of charge from The Cambridge Crystallographic Data Centre via www.ccdc.cam.ac.uk/structures.

### 3.4. Computational Studies

The DFT and TD–DFT calculations were performed by using the Gaussian 16 [50] program at the B3LYP/6–31+G(d,p) level with a polarizable continuum model (PCM) (CH_2_Cl_2_ for **1a**, **2a**, and **3a**; CHCl_3_ for *trans*-**1b**, *trans*-**2b**, and *trans*-**3b**). The starting molecular models for DFT geometry optimizations were built and optimized with MMFF molecular mechanics by using the Spartan ’08 package (Wavefunction, Inc., Irvine, CA, USA). Thirty singlet states were calculated in the TD–DFT calculations. The simulated absorption spectra were visualized by using the SpecDis program [51]. The visualization of the molecular orbitals has been performed using GaussView 6.

## 4. Conclusions

In summary, we have synthesized dibenzo[*d*,*d*′]naphtho[2,3-*b*:6,7-*b*′]disilole and -diphosphole derivatives NDS **3a** and NDP **3b**. The photophysical properties of NDS and NDP were revealed through UV–vis absorption and photoluminescence spectroscopies and theoretical calculations, and were compared with those of the related compounds ever reported in order to clarify the effect of π-extension. For a series of silole-fused compounds, the longest wavelength absorption band was found to be red-shifted in the order benzo[1,2-*b*:4,5-*b*′]disilole < NDS < silolo[3,2-*b*]silole derivatives. In contrast, for a series of phosphole-fused compounds, π-extension from phospholo[3,2-*b*]phosphile to NDP derivatives causes a decrease in the absorption coefficient of the longest wavelength absorption band and induces the red-shift of the second longest wavelength absorption band. Both NDS and NDP exhibit much lower fluorescence quantum yields than their less π-extended homologues. Theoretical calculations revealed that the insertion of a naphthalene unit between two heterole units has great impact on their LUMOs. No distribution on the silicon or phosphorus atom was observed for the LUMOs of NDS **3a** and NDP *trans*-**3b**, while the silicon and phosphorus atoms well contribute to the LUMOs of the less π-extended homologues **1a**, *trans*-**1b**, **2a**, and *trans*-**2b**. These results clearly demonstrate that the aromatic units fused with silole and phosphole units affect the contribution of silicon and phosphorus atoms to the frontier molecular orbitals, which would be informative for designing silole- and/or phosphole-fused polycyclic compounds with desired photophysical properties.

## Figures and Tables

**Table 1:** Photophysical data of silole- and phosphole-fused polycyclic compounds **1**–**3**.

	λ_abs_ (nm)	*ε* (×10^4^·M·cm^−1^)	λ_em_ (nm)	Φ
**3a** *^a^*	347	4.67	387	0.08 (0.30) *^g^*
*cis*-**3b** *^b^*	345, 402	5.27, 0.11	412	0.11 (0.03) *^g^*
*trans*-**3b** *^b^*	345, 402	4.63, 0.12	412	0.11 (0.08) *^g^*
**1a** *^c^*	360	1.20	426	0.58
*trans*-**1b** *^d^*	395	0.69	480	0.98
**2a** *^e^*	322	1.78	370	0.45
*trans*-**2b** *^f^*	330, 383	~3.0, 0.28	420	0.81

*^a^* In CH_2_Cl_2_. *^b^* In CHCl_3_ for absorption and CH_2_Cl_2_ for emission. *^c^* In THF ([29]). *^d^* In CH_2_Cl_2_ ([30]). *^e^* In CH_2_Cl_2_ ([36]). *^f^* In CHCl_3_ ([35]). *^g^* Absolute quantum yield in the solid state in parentheses.

## Data Availability

The data presented in this study are available in the Supplementary Materials.

## Supplementary Materials

The following supporting information can be downloaded at: https://www.mdpi.com/article/10.3390/molecules29184313/s1, Figures S1–S21: ^1^H, ^13^C, and ^31^P NMR spectra for **3** and **5**–**10**; Tables S1 and S2: Crystallographic data for **3a** and *trans*-**3b**; Figures S22–S27: Molecular orbitals for **1**–**3**; Tables S3–S8: Coordinates and absolute energy of the optimized structures for **1**–**3**; Figure S28: Simulated absorption spectra; Figure S29: Natural transition orbital diagrams for absorption and emission; Table S9: The selected absorption of **1**–**3** calculated by the TD–DFT method.
