# Supplementary material for: Synthesis and Properties of Dibenzo-Fused Naphtho[2,3-b:6,7-b′]disilole and Naphtho[2,3-b:6,7-b′]diphosphole
Source: Molecules. 2024 Sep 11;29(18):4313. doi: 10.3390/molecules29184313 (PMC11433746; doi:10.3390/molecules29184313)
Supplement: Supplementary file 1 [file molecules-29-04313-s001.zip › molecules-3173009-supplementary materials.pdf]

Supporting Information

Synthesis and Properties of Dibenzo-fused Naphtho[2,3-*b*:6,7-*b*']disilole and Naphtho[2,3-*b*:6,7-*b*']diphosphole

Suzuho Morishita,[1] Chikara Hayasaka,[1] Keiichi Noguchi,[2] Koji Nakano[1,*]

[1]Department of Organic and Polymer Materials Chemistry, and [2]Instrumentation Analysis Center, Tokyo University of Agriculture and Technology, 2-24-16 Naka-cho, Koganei, Tokyo 184-8588, Japan

e-mail: k_nakano@cc.tuat.ac.jp

Table of Contents

NMR Spectra of Compounds **3** and **5–10** S2−S12

X-ray Analysis S13−S14

DFT and TD-DFT Calculation Results S15−S31

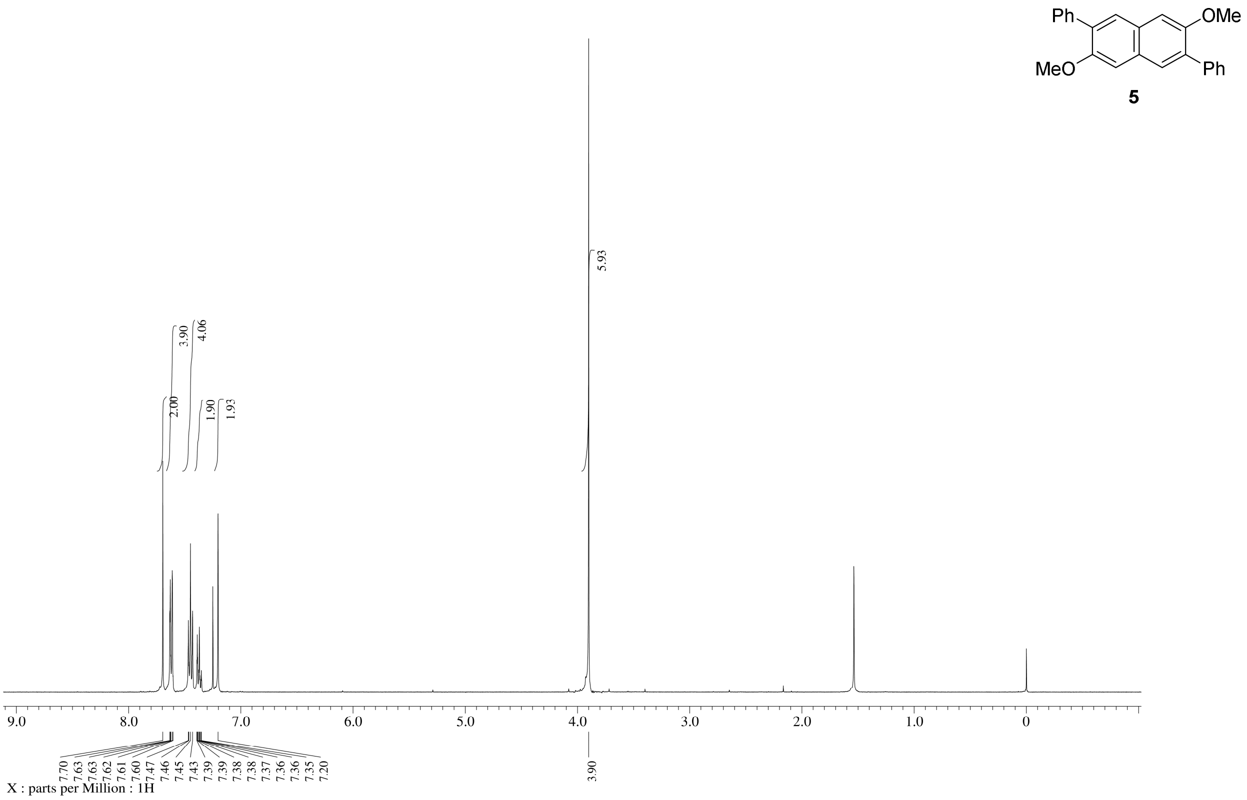

Figure S1. ^{1}H NMR spectrum of **5** (400 MHz, $CDCl_3$).

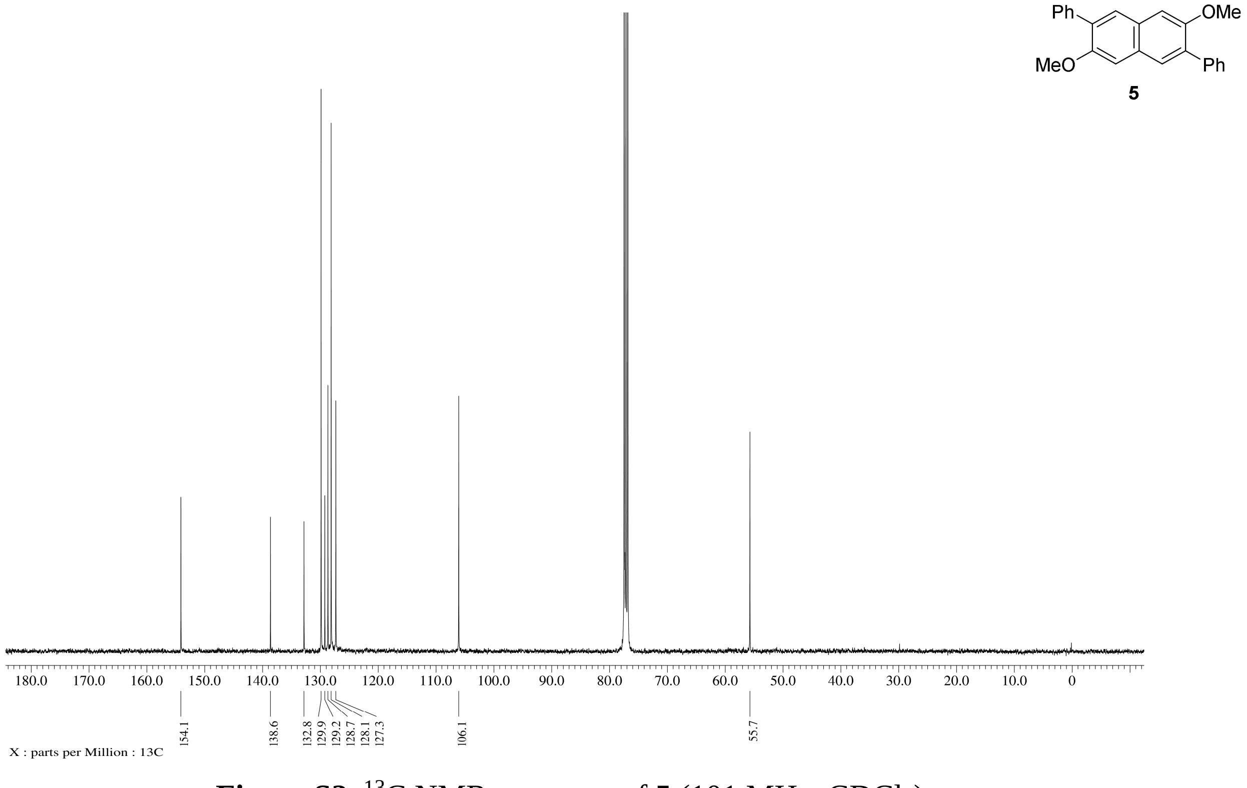

Figure S2. ^{13}C NMR spectrum of **5** (101 MHz, $CDCl_3$).

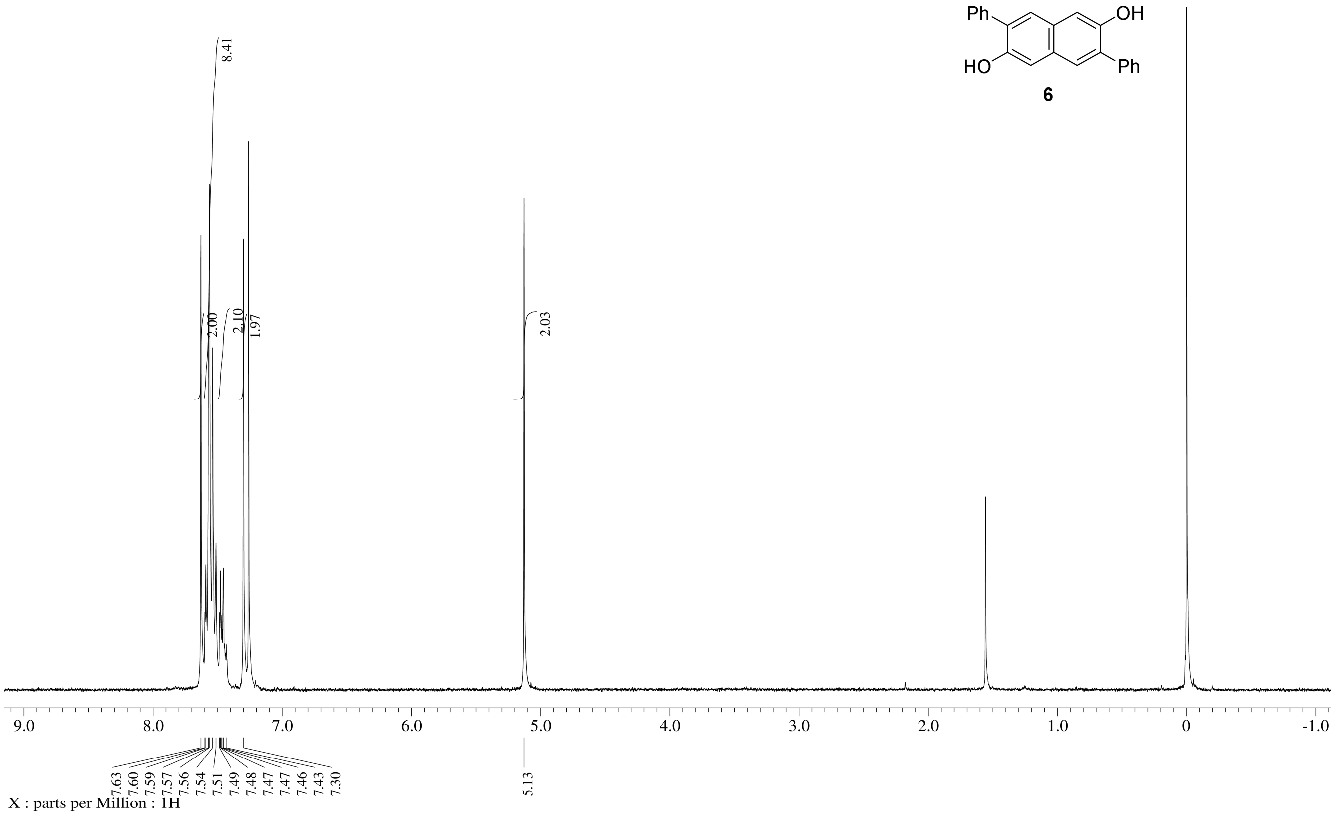

Figure S3. ^{1}H NMR spectrum of **6** (300 MHz, $CDCl_3$).

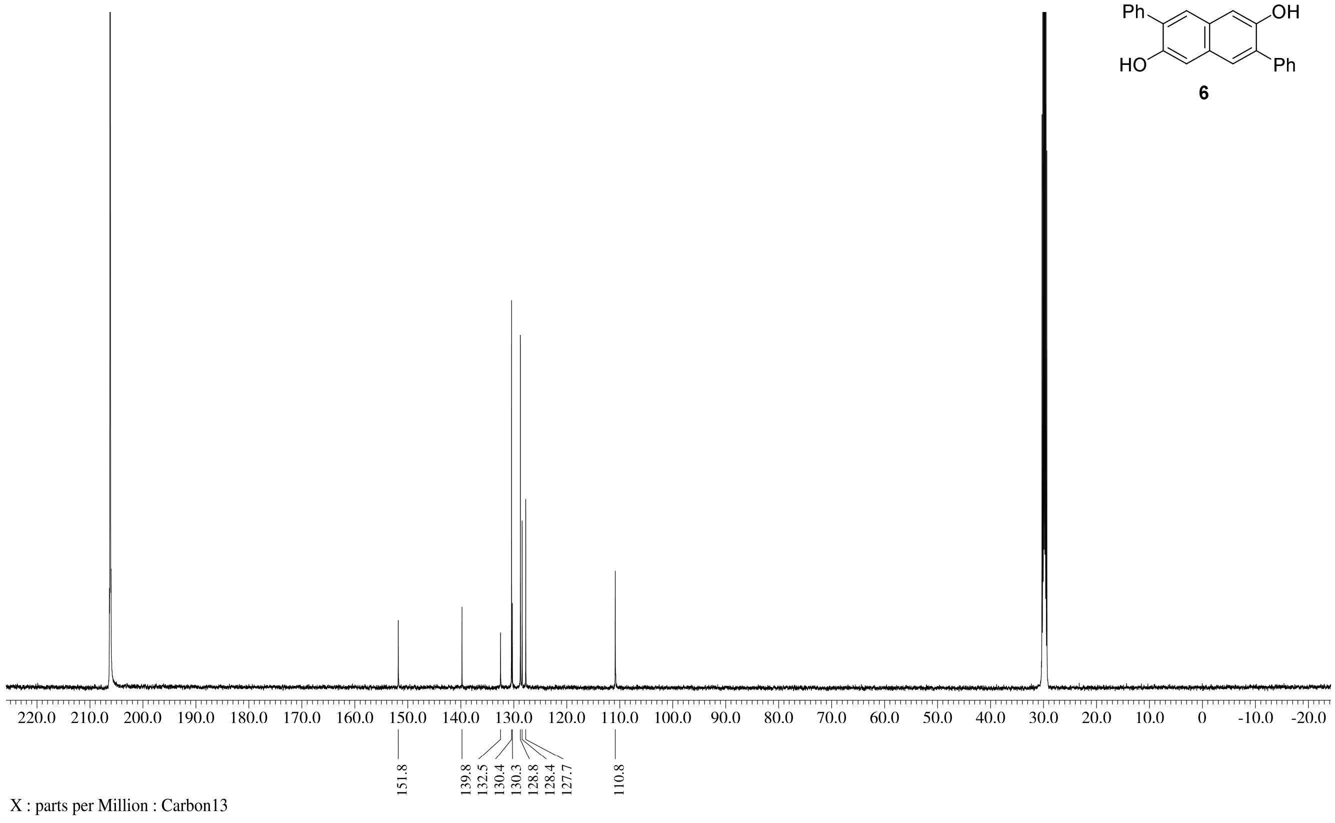

Figure S4. ^{13}C NMR spectrum of **6** (126 MHz, acetone-d_6).

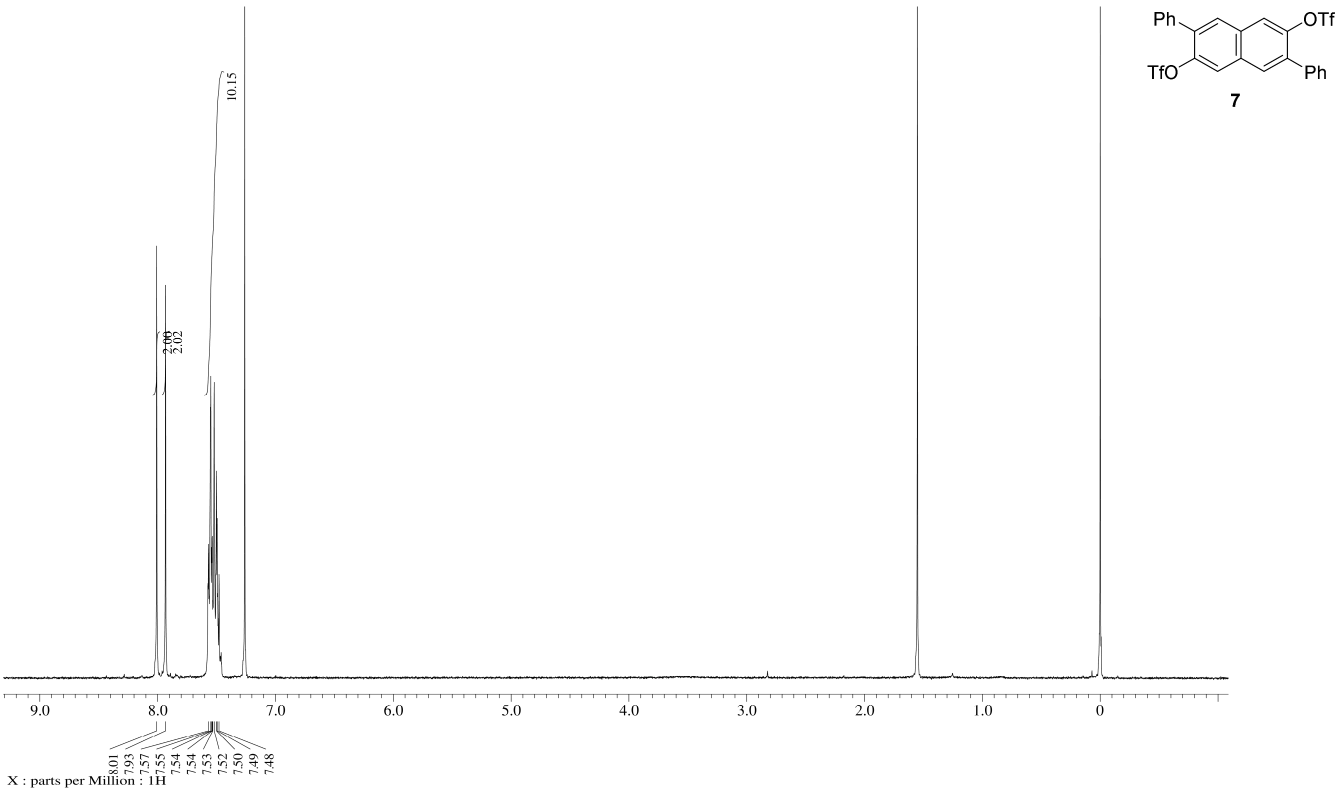

Figure S5. ^{1}H NMR spectrum of **7** (400 MHz, $CDCl_3$).

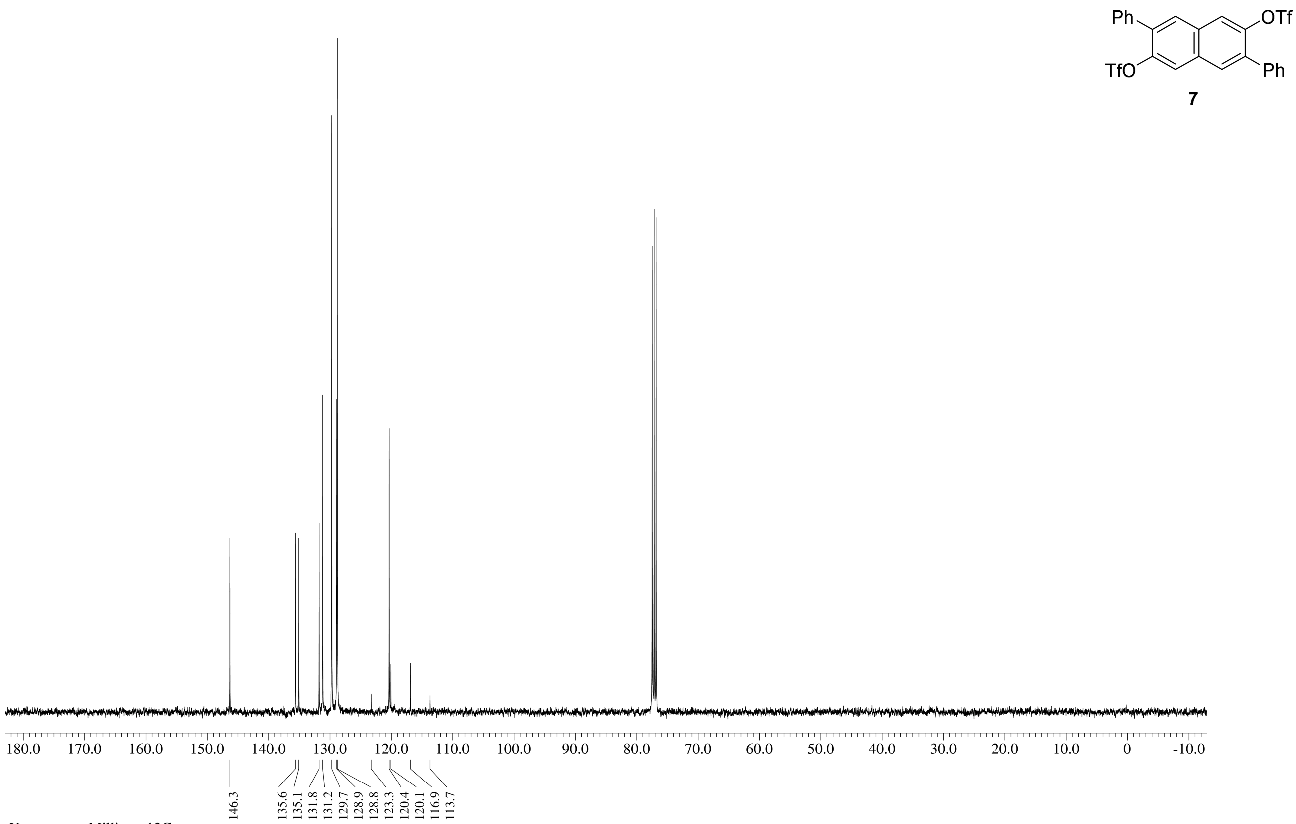

Figure S6. ^{13}C NMR spectrum of **7** (101 MHz, $CDCl_3$).

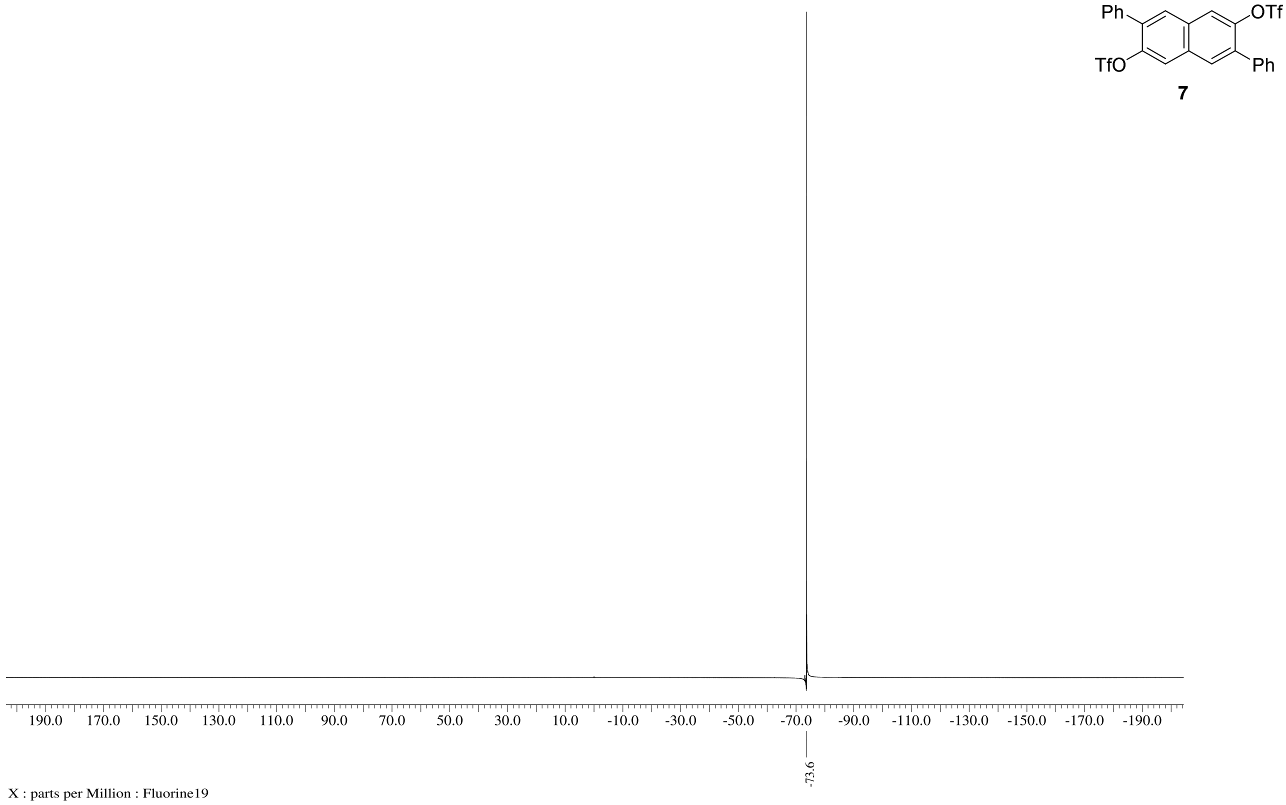

Figure S7. ^{19}F NMR spectrum of **7** (471 MHz, $CDCl_3$).

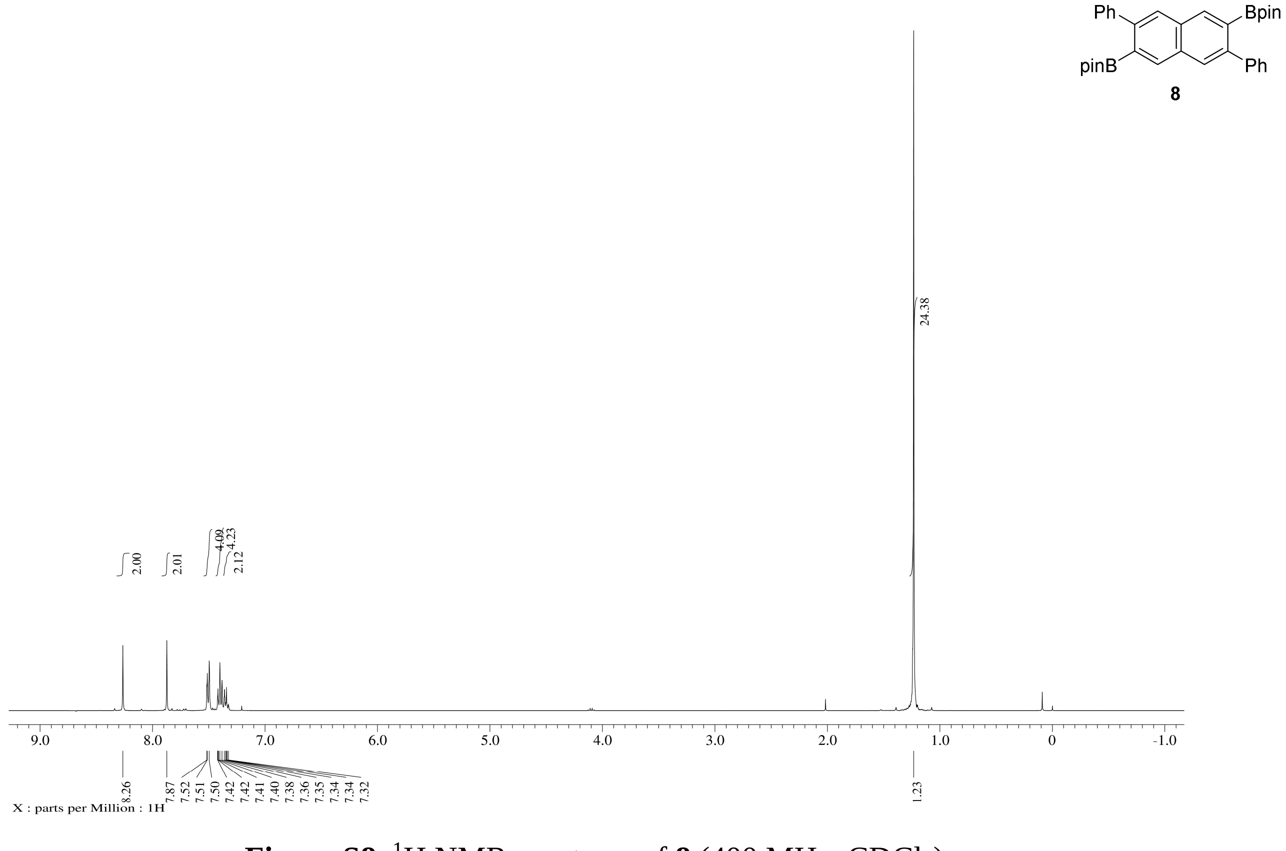

Figure S8. ^{1}H NMR spectrum of **8** (400 MHz, $CDCl_3$).

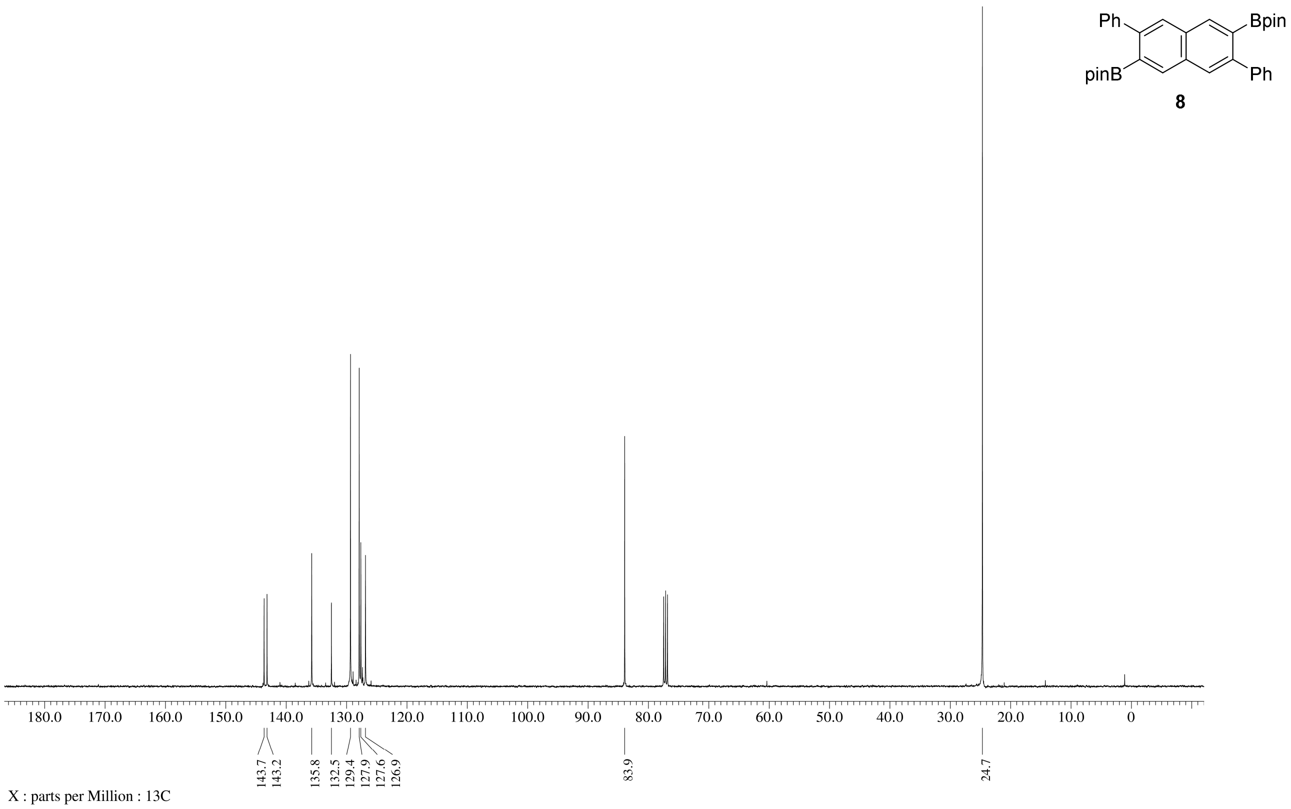

Figure S9. ^{13}C NMR spectrum of **8** (101 MHz, $CDCl_3$).

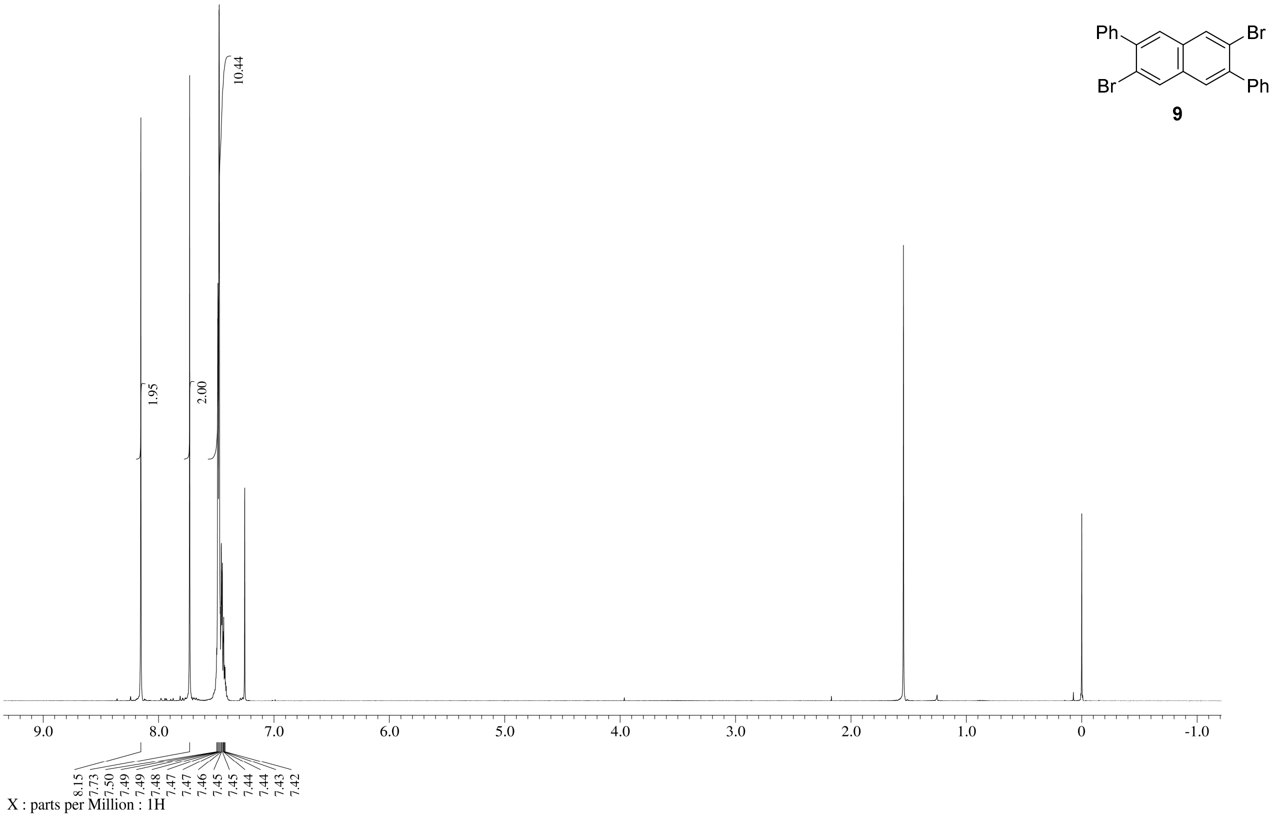

Figure S10. ^{1}H NMR spectrum of **9** (400 MHz, $CDCl_3$).

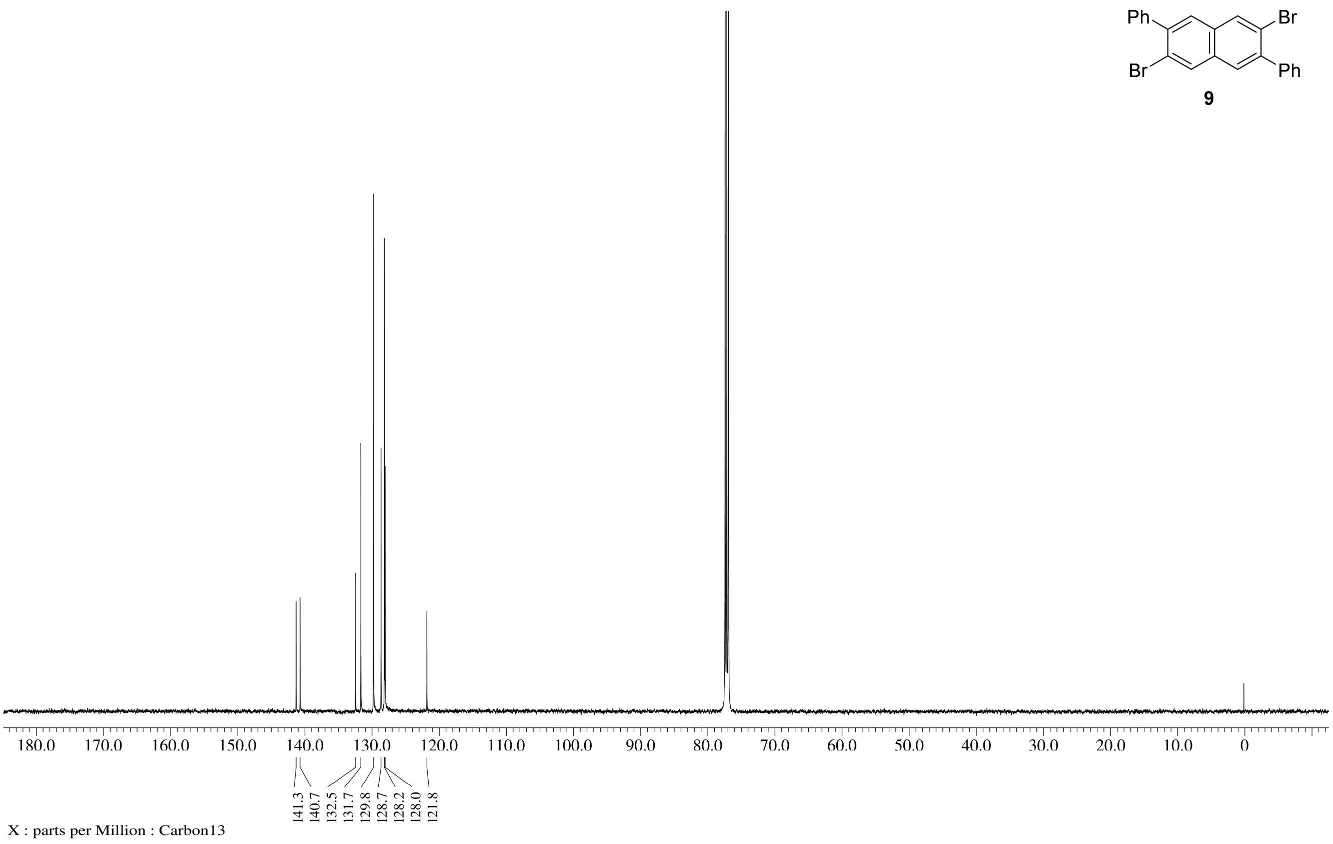

Figure S11. ^{13}C NMR spectrum of **9** (126 MHz, $CDCl_3$).

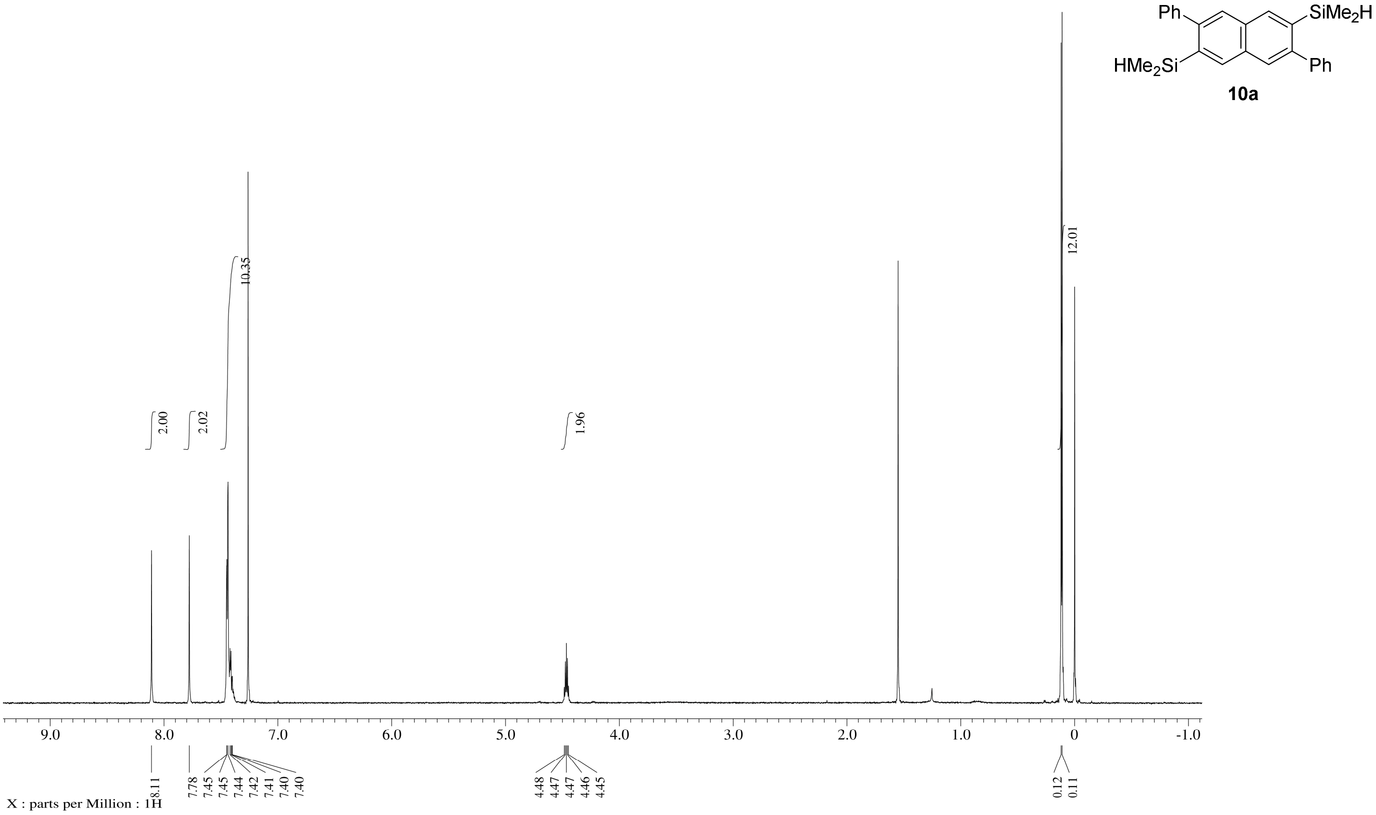

Figure S12. ^{1}H NMR spectrum of **10a** (400 MHz, $CDCl_3$).

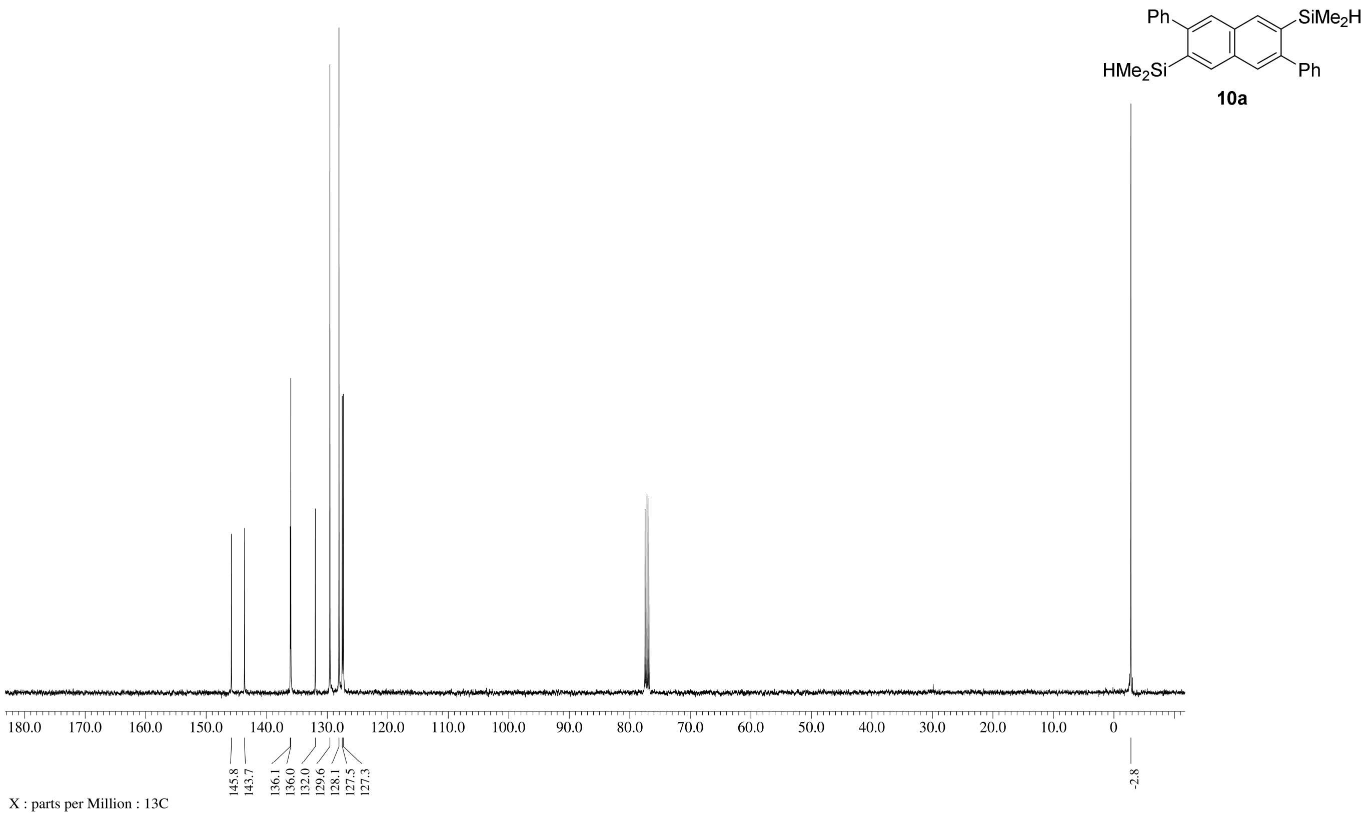

Figure S13. ^{13}C NMR spectrum of **10a** (101 MHz, CDCl$_3$).

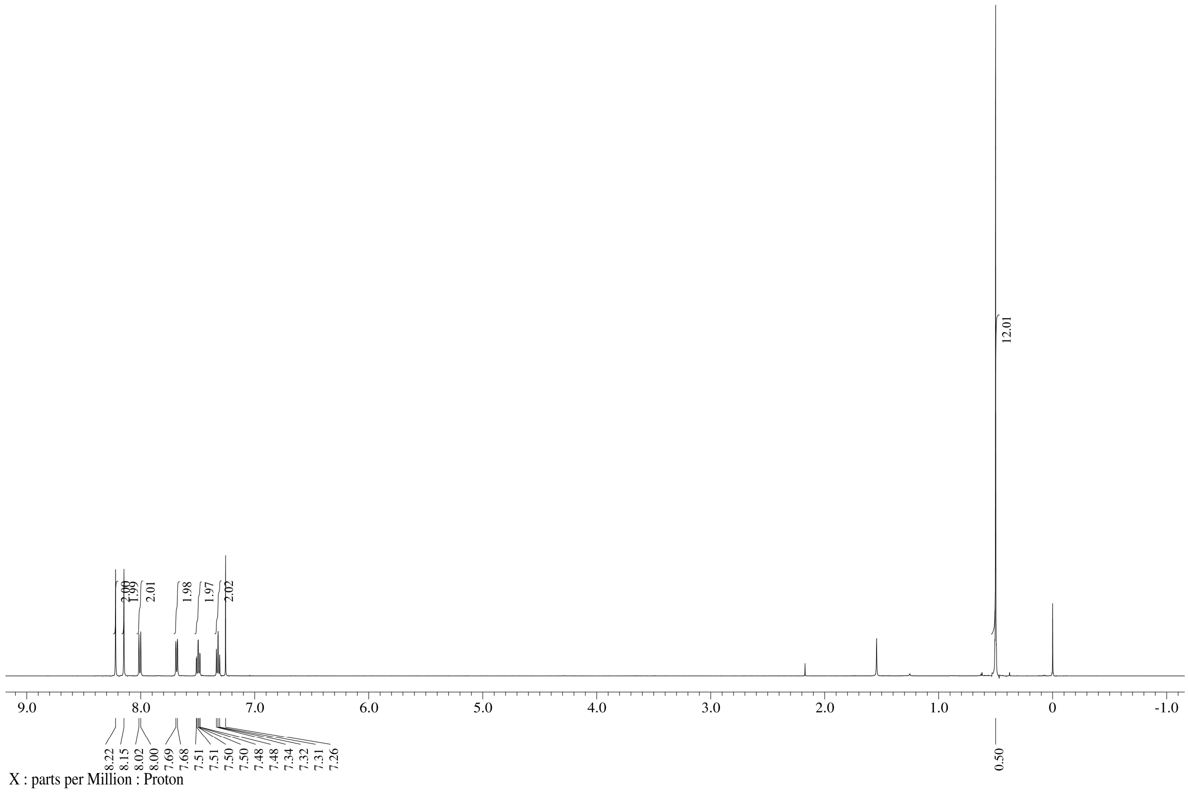

Figure S14. ^{1}H NMR spectrum of **3a** (500 MHz, CDCl$_3$).

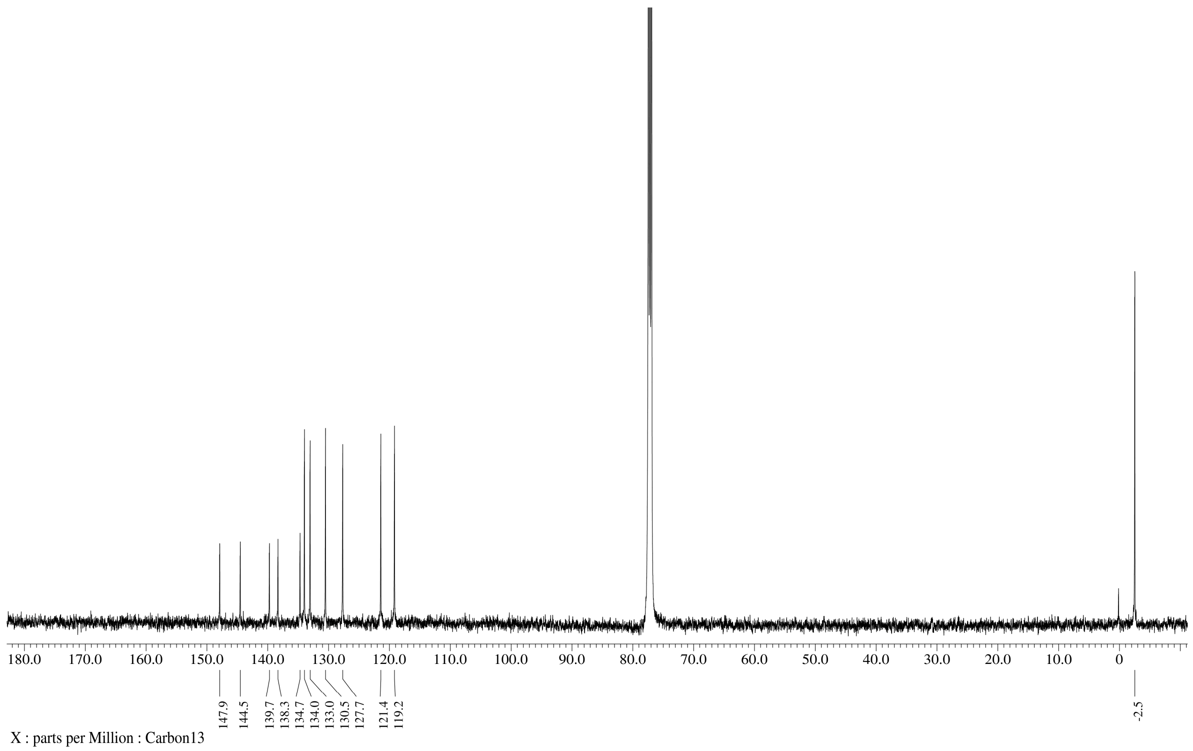

Figure S15. ^{13}C NMR spectrum of **3a** (126 MHz, $CDCl_3$).

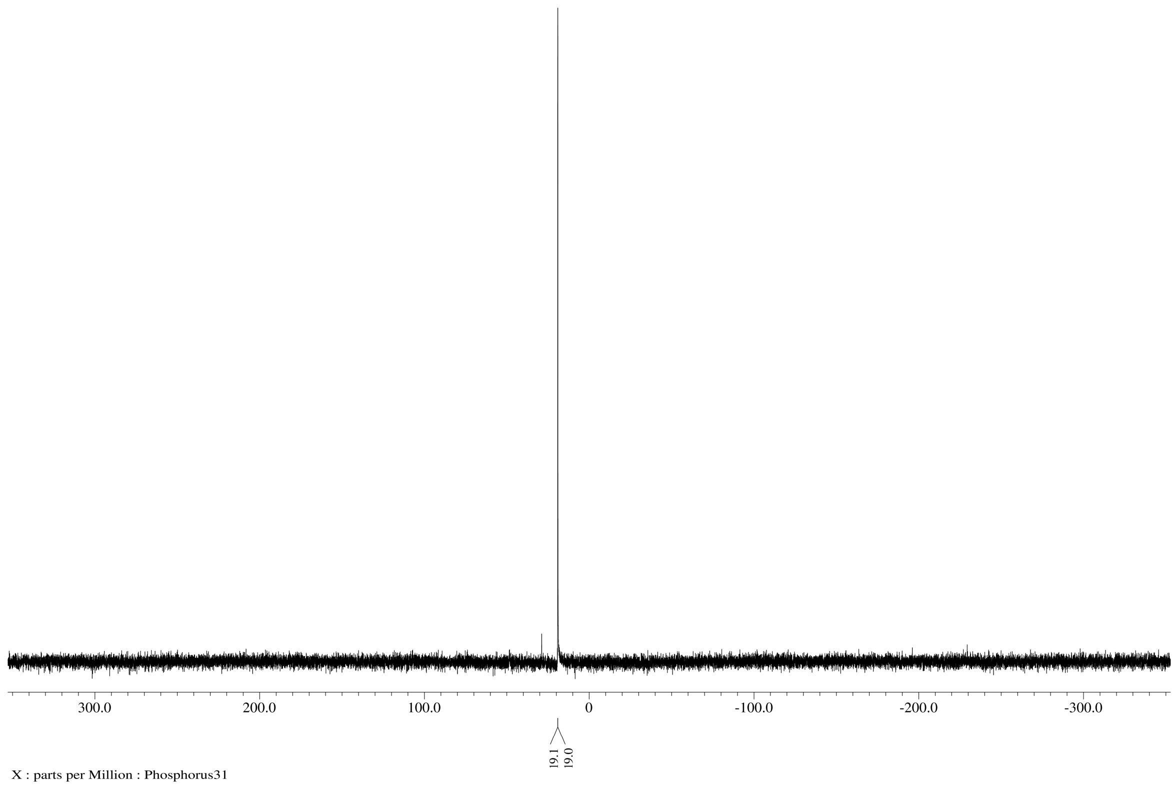

Figure S16. ^{31}P NMR spectrum of **10b** (202 MHz, $CDCl_3$).

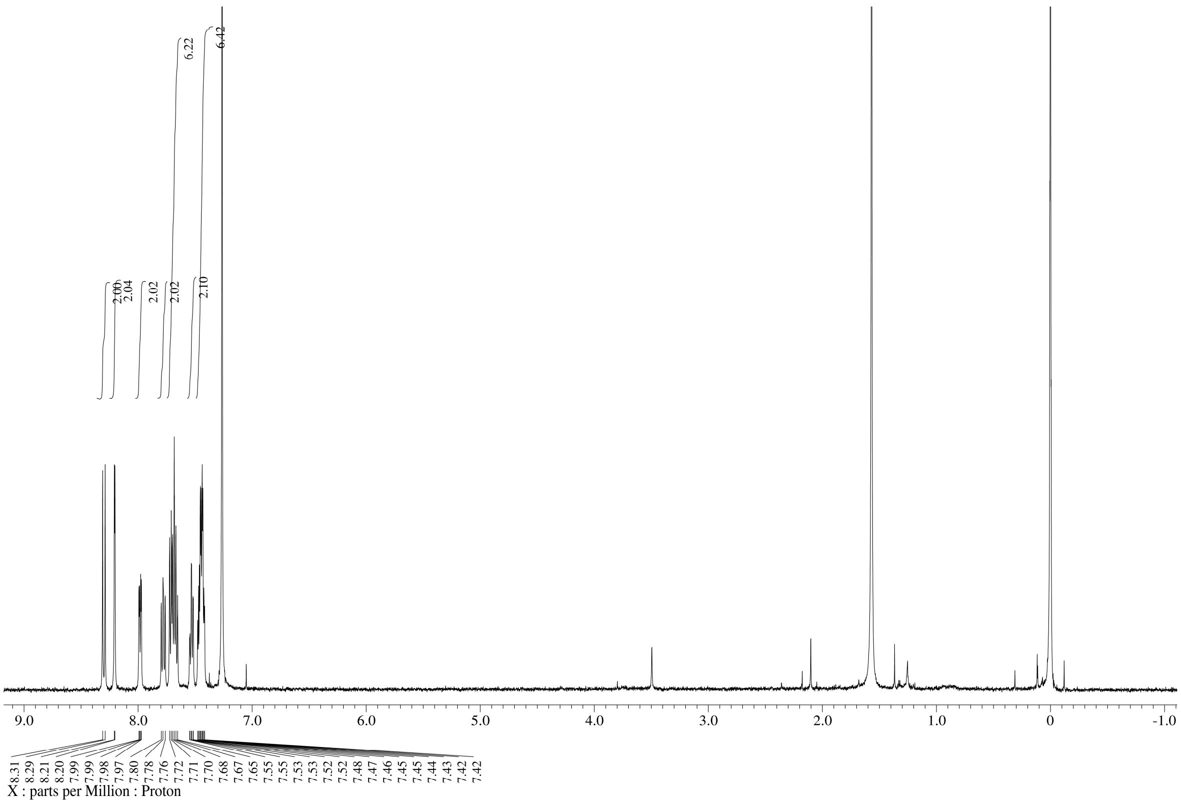

Figure S17. ^{1}H NMR spectrum of *trans*-**3b** (500 MHz, $CDCl_3$).

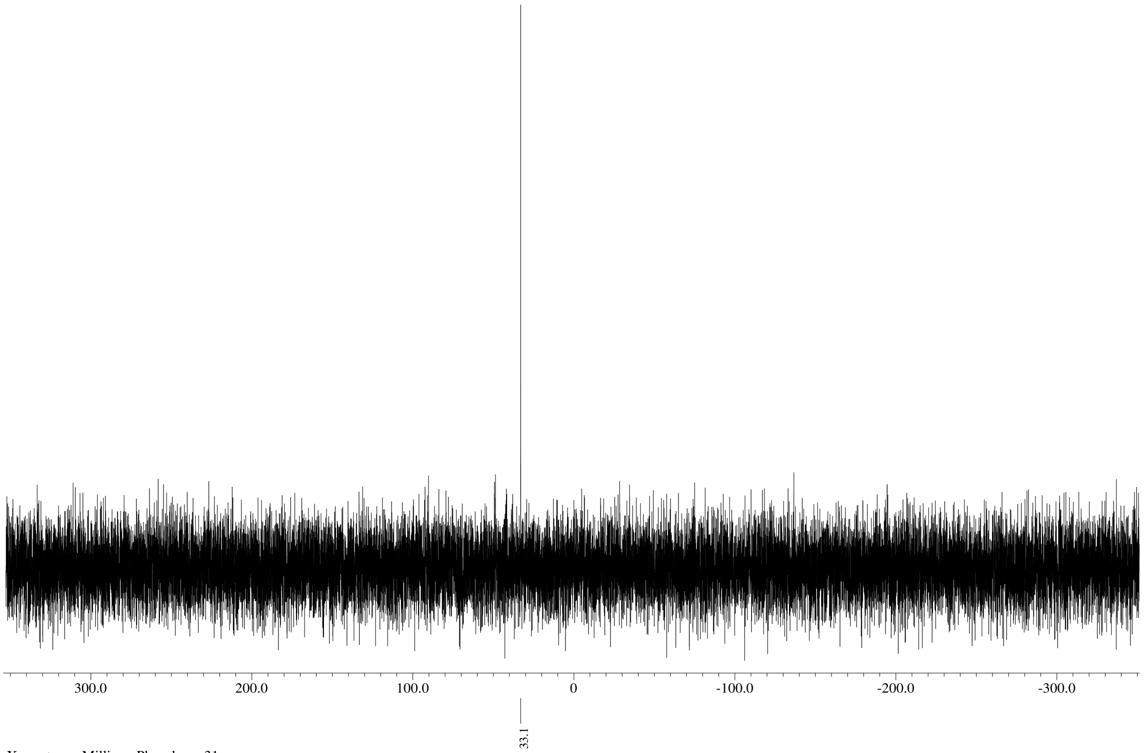

Figure S18. ^{31}P NMR spectrum of *trans*-**3b** (202 MHz, $CDCl_3$).

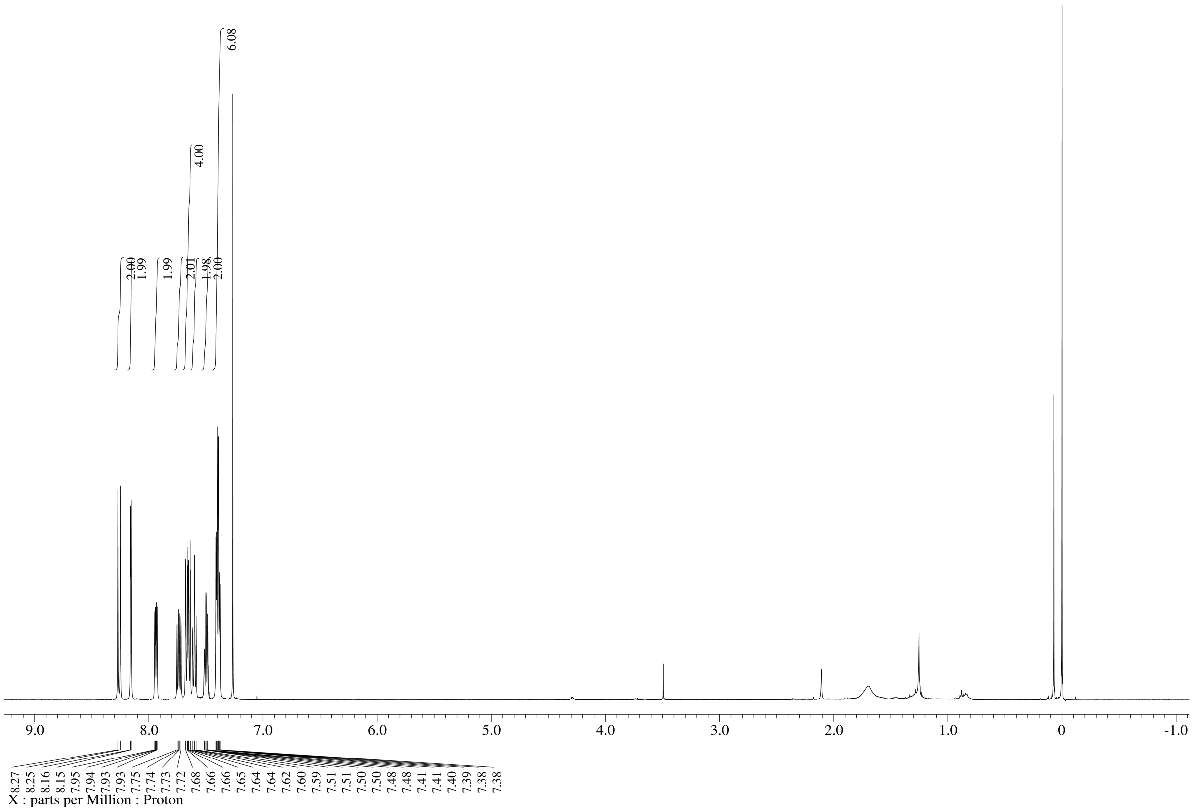

Figure S19. ^{1}H NMR spectrum of *cis*-**3b** (500 MHz, $CDCl_3$).

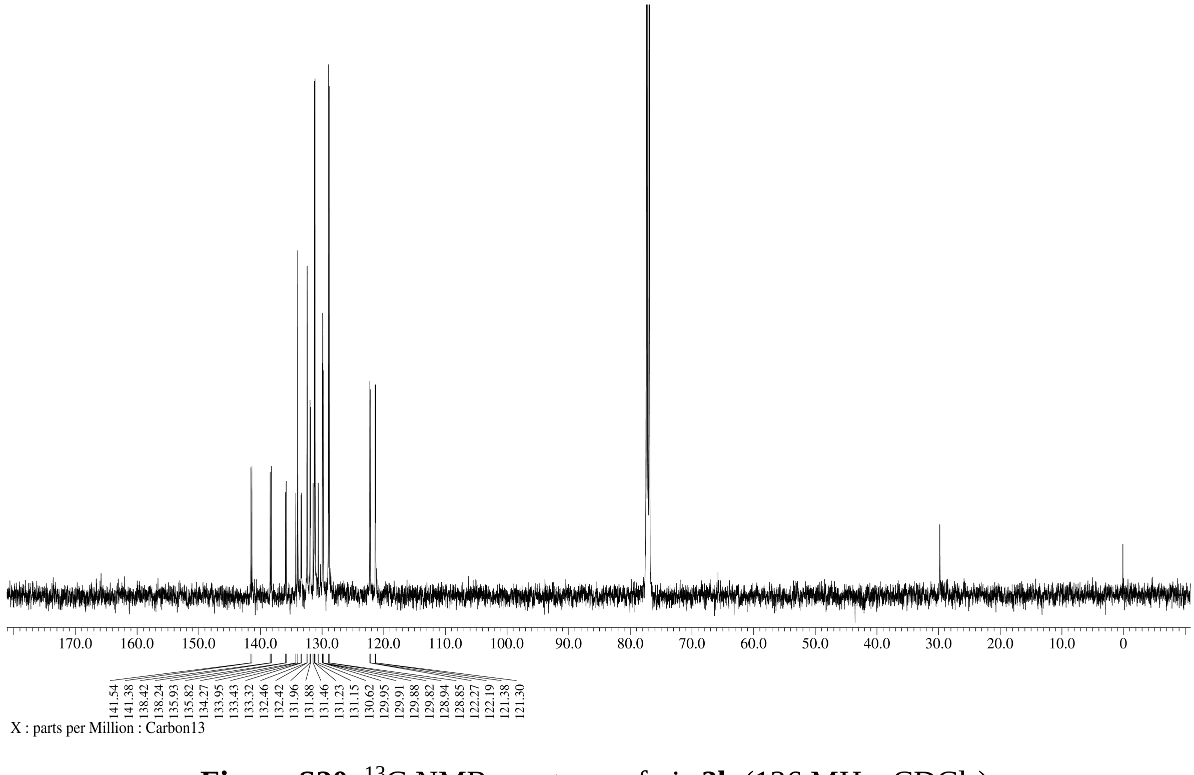

Figure S20. ^{13}C NMR spectrum of *cis*-**3b** (126 MHz, $CDCl_3$).

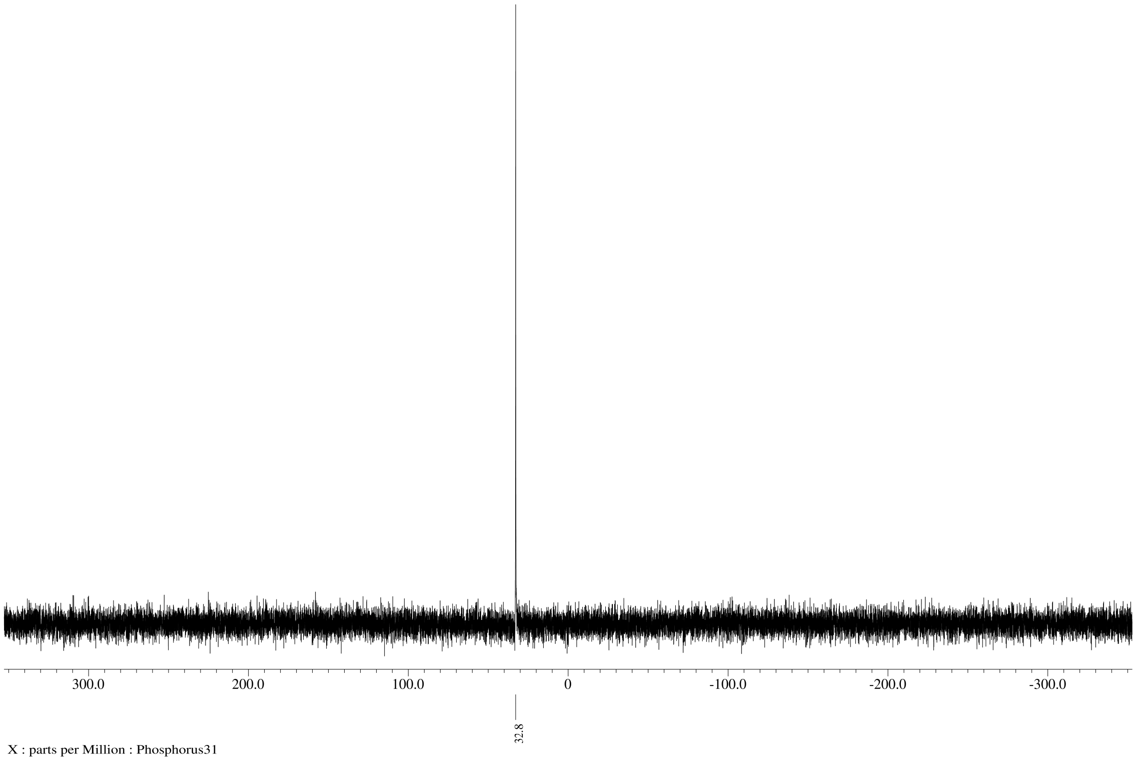

Figure S21. ^{31}P NMR spectrum of *cis*-**3b** (202 MHz, $CDCl_3$).

Table S1. Crystallographic Data and Structure Refinement Details for **3a**

Formula	$C_{26}H_{24}Si_2 \cdot C_4H_8O_2$	
Formula weight	480.73	
Temperature	203(2) K	
Wavelength	1.54187 Å	
Crystal system	monoclinic	
Space group	$P2_1/c$	
Unit cell dimensions	a = 8.00343(18) Å	α = 90°
	b = 12.0224(2) Å	β = 106.8188(12)°
	c = 14.4880(3) Å	γ = 90°
Volume	1334.42(5) $Å^3$	
Z	2	
Density (calculated)	1.196 g/cm^3	
Absorption coefficient	1.389 mm^{-1}	
F(000)	512	
Crystal size	0.40 × 0.30 × 0.20 mm^3	
Theta range for data collection	4.868 to 68.201°	
Index ranges	–9<=h<=9, –14<=k<=14, –17<=l<=17	
Reflections collected	23203	
Independent reflections	2453 [R_{int} = 0.0245]	
Completeness to theta	100.0%	
Max. and min. transmission	0.757 and 0.561	
Refinement method	Full-matrix least-squares on F^2	
Data / restraints / parameters	2453 / 0 / 154	
Goodness-of-fit on F^2	1.111	
Final R indices [I>2sigma(I)]	R_1 = 0.0481, wR_2 = 0.1224	
R indices (all data)	R_1 = 0.0497, wR_2 = 0.1240	
Largest diff. peak and hole	0.374 and –0.301 e/$Å^3$	

Table S2. Crystallographic Data and Structure Refinement Details for *trans*-**3b**

Formula	$C_{17}H_{11}OP{\cdot}CHCl_3$	
Formula weight	381.60	
Temperature	203(2) K	
Wavelength	1.54187 Å	
Crystal system	monoclinic	
Space group	$P2_1/c$	
Unit cell dimensions	a = 7.75254(14) Å	α = 90°
	b = 18.8395(3) Å	β = 101.6418(7)°
	c = 12.3790(2) Å	γ = 90°
Volume	1770.81(6) Å^3	
Z	4	
Density (calculated)	1.431 g/cm^3	
Absorption coefficient	5.541 mm^{-1}	
F(000)	776	
Crystal size	0.60 × 0.25 × 0.20 mm^3	
Theta range for data collection	4.337 to 68.224°	
Index ranges	–9<=h<=9, –22<=k<=22, –14<=l<=14	
Reflections collected	32218	
Independent reflections	3227 [R_{int} = 0.0294]	
Completeness to theta	99.5%	
Max. and min. transmission	0.330 and 0.116	
Refinement method	Full-matrix least-squares on F^2	
Data / restraints / parameters	3227 / 64 / 226	
Goodness-of-fit on F^2	1.051	
Final R indices [I>2sigma(I)]	R_1 = 0.0471, wR_2 = 0.1325	
R indices (all data)	R_1 = 0.0507, wR_2 = 0.1389	
Largest diff. peak and hole	0.616 and –0.308 e/Å^3	

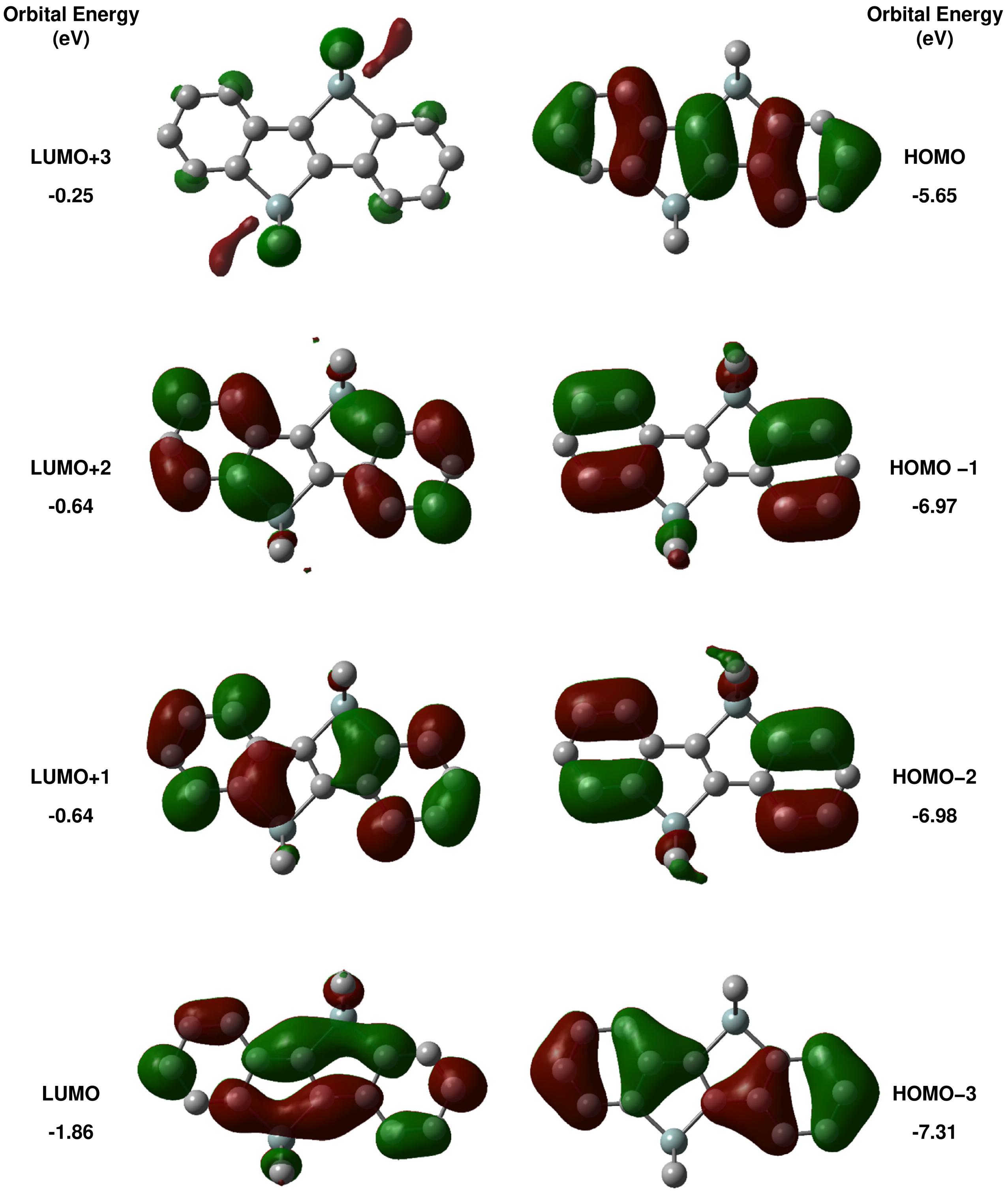

Figure S22. Molecular orbitals of **1a** calculated by DFT method at the B3LYP/6-31+G(d,p) level of theory with PCM (CH_2Cl_2).

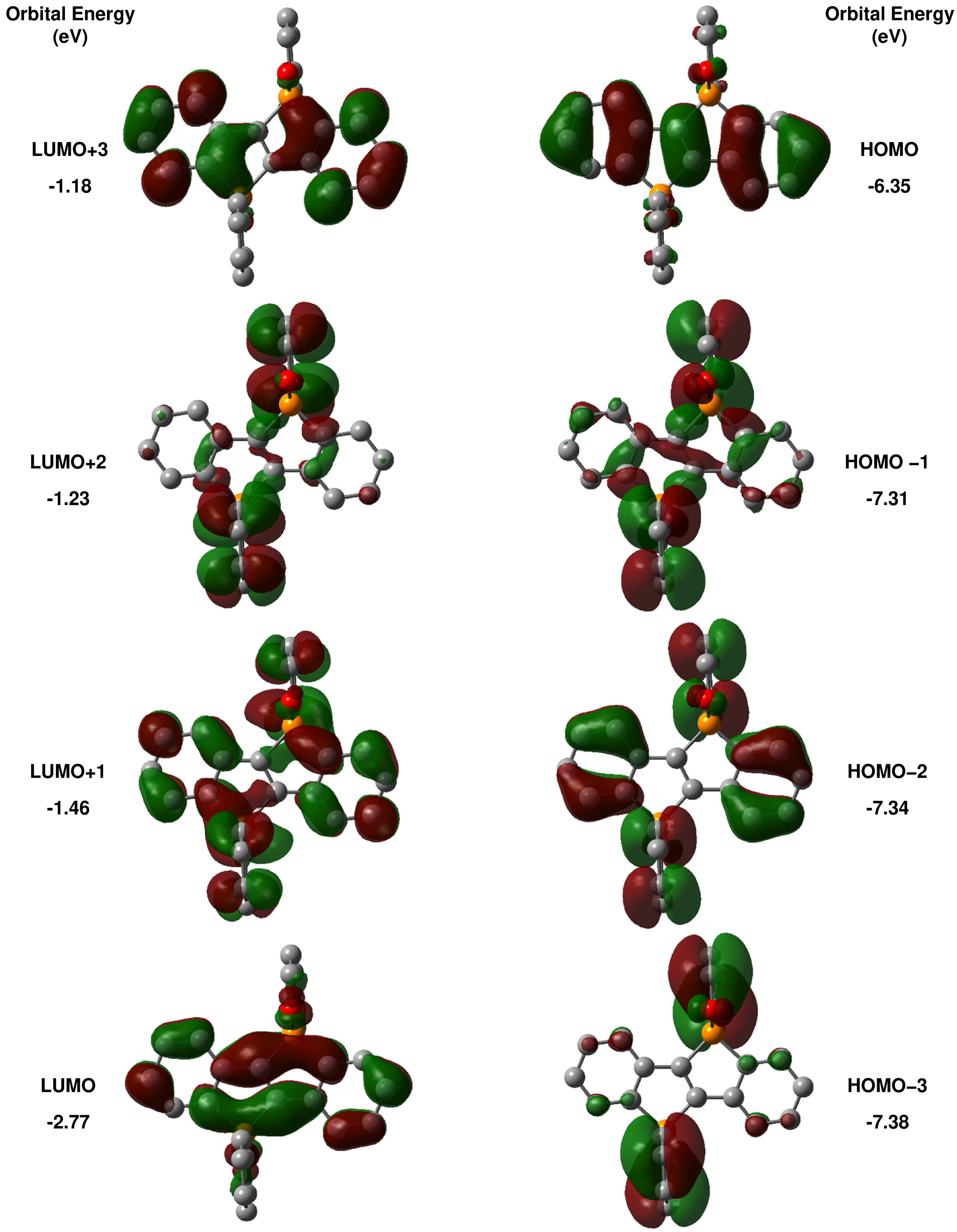

Figure S23. Molecular orbitals of *trans*-**1b** calculated by DFT method at the B3LYP/6-31+G(d,p) level of theory in conjunction with PCM ($CHCl_3$).

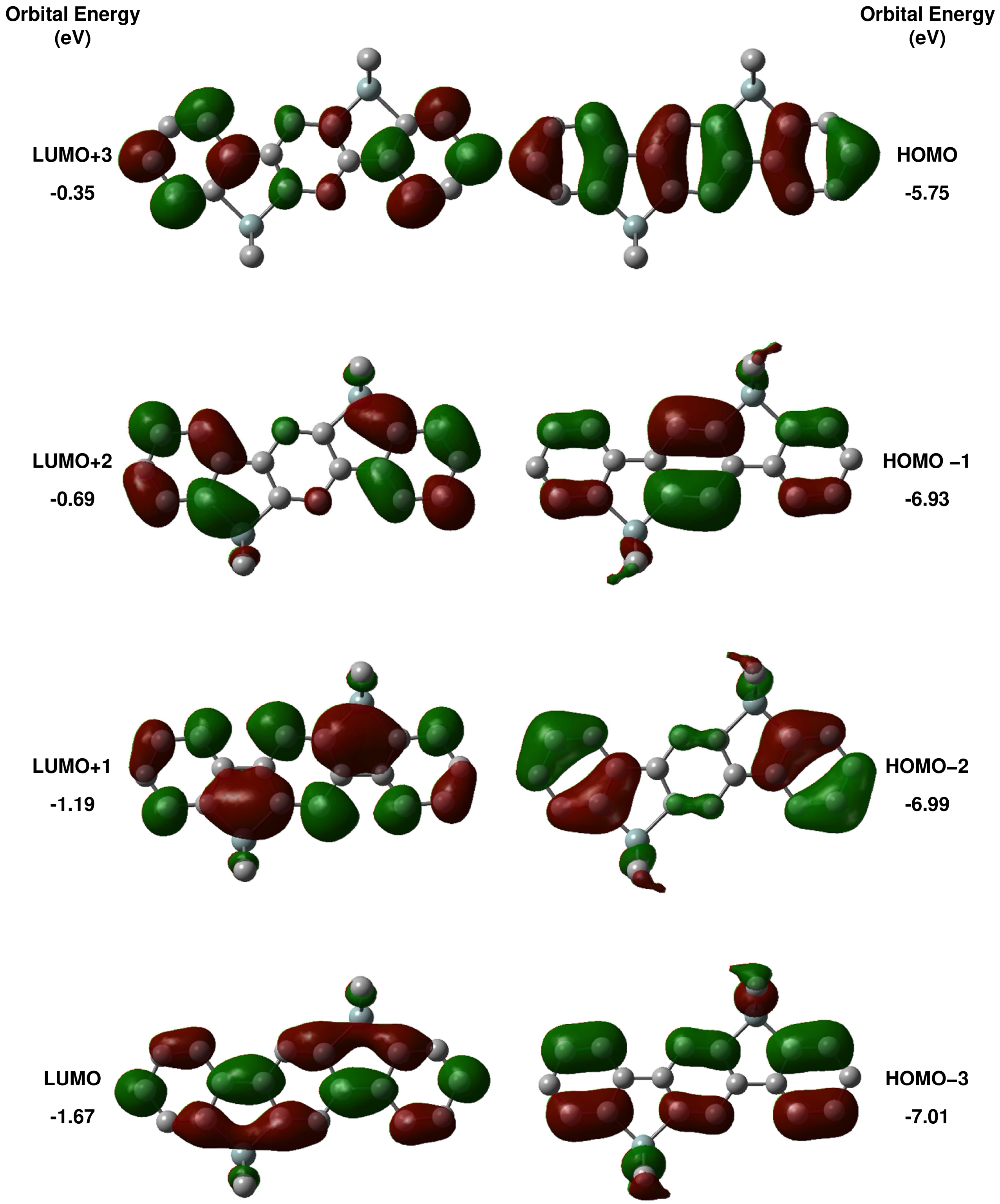

Figure S24. Molecular orbitals of **2a** calculated by DFT method at the B3LYP/6-31+G(d,p) level of theory with PCM (CH_2Cl_2).

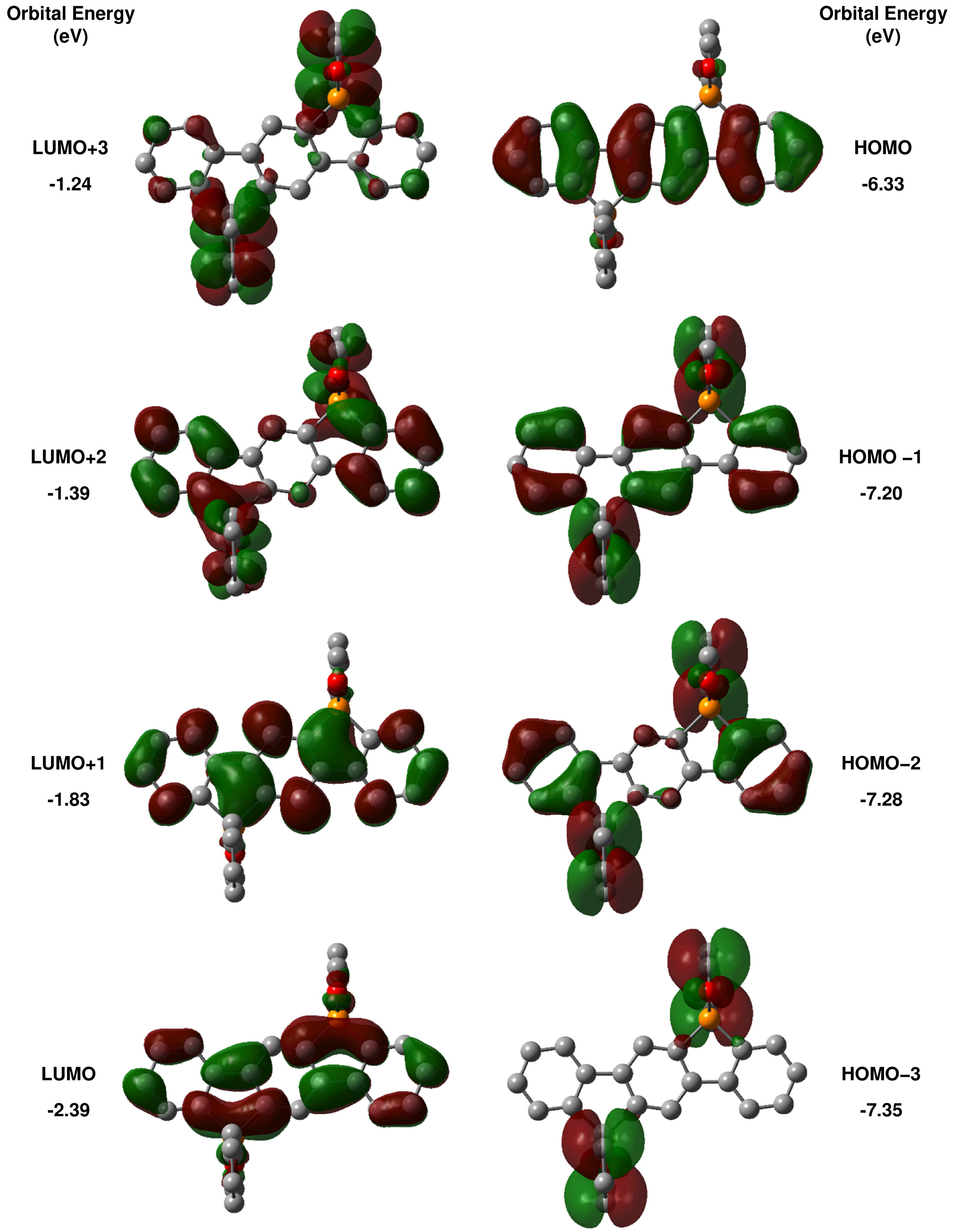

Figure S25. Molecular orbitals of *trans*-**2b** calculated by DFT method at the B3LYP/6-31+G(d,p) level of theory with PCM ($CHCl_3$).

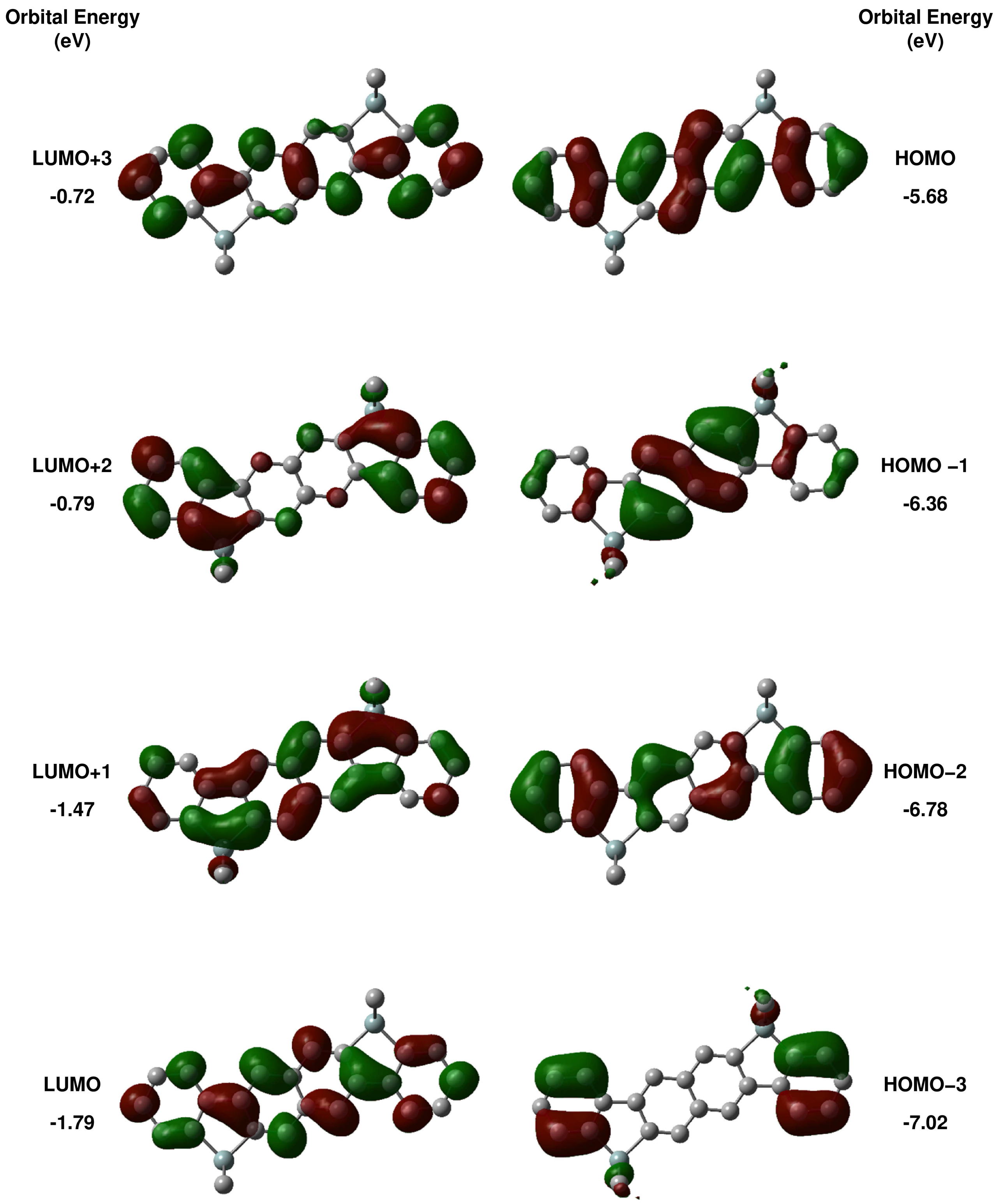

Figure S26. Molecular orbitals of **3a** calculated by DFT method at the B3LYP/6-31+G(d,p) level of theory with PCM (CH_2Cl_2).

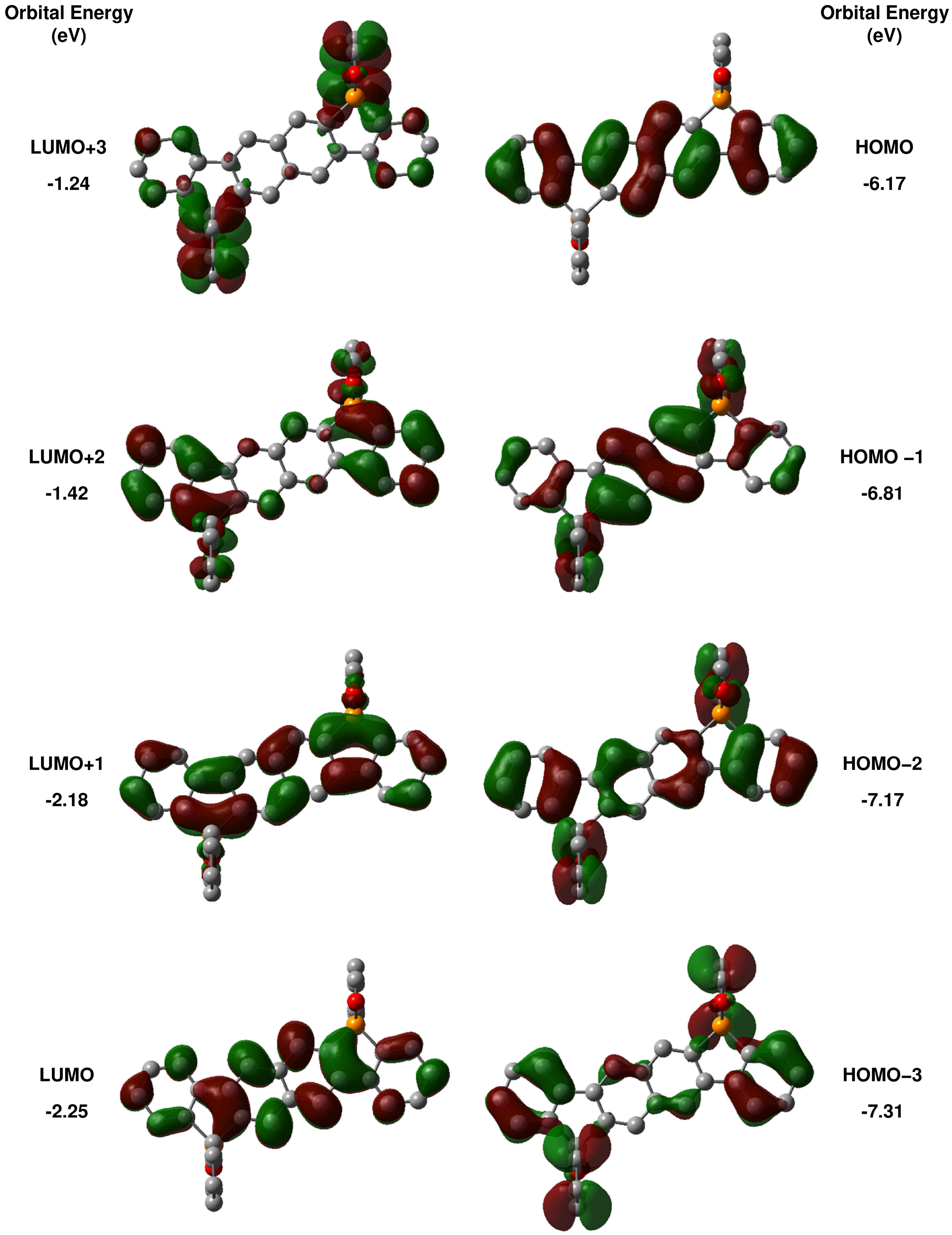

Figure S27. Molecular orbitals of *trans*-**3b** calculated by DFT method at the B3LYP/6-31+G(d,p) level of theory with PCM ($CHCl_3$).

Table S3. Coordinates (Å) and Absolute Energy of the Optimized Structure for **1a**[a]

atom	x	y	z	atom	x	y	z
C	-0.0007073	-2.4828878	0.0000000	H	-3.6893541	3.7390811	-0.0000000
C	2.6614888	-3.3866870	-0.0000000	H	-1.8182929	5.3723943	-0.0000001
C	1.0758864	-1.5489459	0.0000000	Si	-1.6248068	-1.5115824	0.0000000
C	0.2803984	-3.8493444	-0.0000000	Si	1.6248068	1.5115824	0.0000000
C	1.6092266	-4.3062413	-0.0000000	C	-2.6614888	-1.7978092	1.5541868
C	2.3999288	-2.0108368	-0.0000000	H	-2.0667447	-1.6324524	2.4584896
H	-0.5279922	-4.5776663	-0.0000000	H	-3.0402358	-2.8262064	1.5783919
H	1.8182929	-5.3723943	-0.0000001	H	-3.5234915	-1.1219513	1.5842307
H	3.2274880	-1.3060937	0.0000000	C	-2.6614888	-1.7978092	-1.5541867
H	3.6893541	-3.7390811	-0.0000000	H	-3.0402358	-2.8262064	-1.5783919
C	0.6750098	-0.1282487	0.0000000	H	-2.0667448	-1.6324523	-2.4584896
C	-0.6750098	0.1282487	0.0000000	H	-3.5234916	-1.1219513	-1.5842305
C	-1.0758864	1.5489459	-0.0000000	C	2.6614888	1.7978092	-1.5541867
C	-1.6092266	4.3062413	0.0000000	H	3.5234916	1.1219513	-1.5842305
C	-2.3999288	2.0108368	0.0000000	H	3.0402358	2.8262064	-1.5783919
C	0.0007073	2.4828878	-0.0000000	H	2.0667448	1.6324523	-2.4584896
C	-0.2803984	3.8493444	-0.0000000	C	2.6614888	1.7978092	1.5541868
C	-2.6614888	3.3866870	0.0000000	H	2.0667447	1.6324524	2.4584896
H	-3.2274880	1.3060937	-0.0000000	H	3.0402358	2.8262064	1.5783919
H	0.5279922	4.5776663	-0.0000000	H	3.5234915	1.1219513	1.5842307
absolute energy *E* (B3LYP): -1277.08188312 au							

[a]Calculated by DFT method [B3LYP/6-31+G(d,p); PCM (CH_2Cl_2)]

Table S4. Coordinates (Å) and Absolute Energy of the Optimized Structure for *trans*-**1b**[a]

atom	x	y	z	atom	x	y	z
C	0.3351075	2.2090391	0.9166055	O	1.1704589	-2.6810165	1.4785266
C	0.7590566	2.0878531	3.6635871	O	-1.1704589	2.6810165	-1.4785266
C	0.3392121	0.9557930	1.5756421	C	1.5710038	2.3247882	-1.7788717
C	0.5343139	3.3880315	1.6231683	C	3.8910900	2.9073104	-3.2291734
C	0.7449824	3.3227614	3.0104798	C	1.5037141	3.1544217	-2.9067897
C	0.5580151	0.8960030	2.9535803	C	2.8046247	1.7873068	-1.3773055
H	0.5271199	4.3489537	1.1168424	C	3.9602082	2.0783926	-2.1031778
H	0.8980338	4.2368487	3.5755981	C	2.6649144	3.4445183	-3.6295738
H	0.5755781	-0.0583850	3.4703724	H	0.5463069	3.5666845	-3.2094342
H	0.9272799	2.0478109	4.7354536	H	2.8679694	1.1443835	-0.5037721
C	0.0943881	-0.1613923	0.6533155	H	4.9122573	1.6601205	-1.7908359
C	-0.0943881	0.1613923	-0.6533155	H	2.6094256	4.0886273	-4.5020739
C	-0.3392121	-0.9557930	-1.5756421	H	4.7923866	3.1325763	-3.7916584
C	-0.7449824	-3.3227614	-3.0104798	C	-1.5710038	-2.3247882	1.7788717
C	-0.5580151	-0.8960030	-2.9535803	C	-3.8910900	-2.9073104	3.2291734
C	-0.3351075	-2.2090391	-0.9166055	C	-1.5037141	-3.1544217	2.9067897
C	-0.5343139	-3.3880315	-1.6231683	C	-2.8046247	-1.7873068	1.3773055
C	-0.7590566	-2.0878531	-3.6635871	C	-3.9602082	-2.0783926	2.1031778
H	-0.5755781	0.0583850	-3.4703724	C	-2.6649144	-3.4445183	3.6295738
H	-0.5271199	-4.3489537	-1.1168424	H	-0.5463069	-3.5666845	3.2094342
H	-0.9272799	-2.0478109	-4.7354536	H	-2.8679694	-1.1443835	0.5037721
H	-0.8980338	-4.2368487	-3.5755981	H	-4.9122573	-1.6601205	1.7908359
P	0.0241638	1.9796611	-0.8748611	H	-2.6094256	-4.0886273	4.5020739
P	-0.0241638	-1.9796611	0.8748611	H	-4.7923866	-3.1325763	3.7916584

absolute energy *E* (B3LYP): -1834.89278128 au

[a]Calculated by DFT method [B3LYP/6-31+G(d,p); PCM ($CHCl_3$)]

Table S5. Coordinates (Å) and Absolute Energy of the Optimized Structure for **2a**[a]

atom	x	y	z	atom	x	y	z
H	-0.1877878	2.4838781	0.0000000	C	-3.8767142	-3.4940023	0.0000000
C	-0.1052560	1.3994565	0.0000000	H	-1.7290455	-3.5662033	0.0000000
C	0.1052560	-1.3994565	0.0000000	H	-5.7779311	-0.6728935	0.0000000
C	1.1625745	0.7953591	0.0000000	H	-3.9778426	-4.5758154	0.0000000
C	-1.2656020	0.6209303	0.0000000	H	-6.0064087	-3.1390631	0.0000000
C	-1.1625745	-0.7953591	0.0000000	Si	-3.0896743	1.1163515	0.0000000
C	1.2656020	-0.6209303	0.0000000	Si	3.0896743	-1.1163515	0.0000000
H	0.1877878	-2.4838781	0.0000000	C	3.6167311	-2.0576682	1.5503915
C	2.4609595	1.5240937	0.0000000	H	3.3152628	-1.5240432	2.4576466
C	5.0189443	2.6861039	0.0000000	H	4.7051257	-2.1857436	1.5745456
C	2.6018922	2.9198402	0.0000000	H	3.1615727	-3.0546093	1.5727764
C	3.6167311	0.6976288	0.0000000	C	3.6167311	-2.0576682	-1.5503915
C	4.8830974	1.2915831	0.0000000	H	4.7051257	-2.1857436	-1.5745456
C	3.8767142	3.4940023	0.0000000	H	3.3152628	-1.5240432	-2.4576466
H	1.7290455	3.5662033	0.0000000	H	3.1615727	-3.0546093	-1.5727764
H	5.7779311	0.6728935	0.0000000	C	-3.6167311	2.0576682	1.5503915
H	3.9778426	4.5758154	0.0000000	H	-3.3152628	1.5240432	2.4576466
H	6.0064087	3.1390631	0.0000000	H	-4.7051257	2.1857436	1.5745456
C	-2.4609595	-1.5240937	0.0000000	H	-3.1615727	3.0546093	1.5727764
C	-5.0189443	-2.6861039	0.0000000	C	-3.6167311	2.0576682	-1.5503915
C	-2.6018922	-2.9198402	0.0000000	H	-3.1615727	3.0546093	-1.5727764
C	-3.6167311	-0.6976288	0.0000000	H	-4.7051257	2.1857436	-1.5745456
C	-4.8830974	-1.2915831	0.0000000	H	-3.3152628	1.5240432	-2.4576466

absolute energy *E* (B3LYP): -1430.74877415 au

[a]Calculated by DFT method [B3LYP/6-31+G(d,p); PCM (CH_2Cl_2)]

Table S6. Coordinates (Å) and Absolute Energy of the Optimized Structure for *trans*-**2b**[a]

atom	x	y	z	atom	x	y	z
H	0.8156914	2.0392329	1.2014500	H	4.7986245	-4.7365826	-0.0630269
C	0.4550162	1.1585418	0.6787257	P	2.9920328	-0.1699297	1.1277597
C	-0.4550162	-1.1585418	-0.6787257	P	-2.9920328	0.1699297	-1.1277597
C	-0.8428423	1.1155738	0.1529079	C	-4.0841643	-0.9161928	-0.1471871
C	1.2789060	0.0475056	0.5186550	C	-5.7920694	-2.6182877	1.2741320
C	0.8428423	-1.1155738	-0.1529079	C	-4.0621047	-0.9237837	1.2569299
C	-1.2789060	-0.0475056	-0.518655	C	-4.9644835	-1.7628529	-0.8348167
H	-0.8156914	-2.0392329	-1.201450	C	-5.8171707	-2.6122061	-0.1228844
C	-1.8610675	2.1913329	0.2059847	C	-4.9139719	-1.7735898	1.9636366
C	-3.9839649	4.0201994	0.1023192	H	-3.3850990	-0.2706101	1.8006701
C	-1.7227778	3.4500392	0.8000454	H	-4.9758238	-1.7490928	-1.9200907
C	-3.0750292	1.8547264	-0.4315511	H	-6.4982130	-3.2658327	-0.6597763
C	-4.1330594	2.7594791	-0.4882059	H	-4.8935679	-1.7769014	3.0492844
C	-2.7861685	4.3570707	0.7431362	H	-6.4543841	-3.2782209	1.8267162
H	-0.8017057	3.7302579	1.3019319	C	4.0841643	0.9161928	0.1471871
H	-5.0622128	2.4949117	-0.9846856	C	5.7920694	2.6182877	-1.2741320
H	-2.6781603	5.3352970	1.2019710	C	4.0621048	0.9237836	-1.2569299
H	-4.7986246	4.7365826	0.0630269	C	4.9644834	1.7628530	0.8348167
C	1.8610675	-2.1913329	-0.2059847	C	5.8171707	2.6122062	0.1228844
C	3.9839649	-4.0201994	-0.1023191	C	4.9139720	1.7735898	-1.9636366
C	1.7227778	-3.4500392	-0.8000454	H	3.3850992	0.2706099	-1.8006701
C	3.0750292	-1.8547264	0.4315512	H	4.9758237	1.7490929	1.9200907
C	4.1330594	-2.7594791	0.4882059	H	6.4982129	3.2658328	0.6597763
C	2.7861685	-4.3570707	-0.7431362	H	4.8935681	1.7769013	-3.0492844
H	0.8017057	-3.7302579	-1.3019319	H	6.4543841	3.2782208	-1.8267163
H	5.0622128	-2.4949117	0.9846856	O	3.2185733	-0.0076659	2.6141087
H	2.6781603	-5.3352970	-1.2019710	O	-3.2185733	0.0076659	-2.6141087
absolute energy *E* (B3LYP): -1988.56406537 au							

[a]Calculated by DFT method [B3LYP/6-31+G(d,p); PCM ($CHCl_3$)]

Table S7. Coordinates (Å) and Absolute Energy of the Optimized Structure for **3a**[a]

atom	x	y	z	atom	x	y	z
C	-0.0139342	-0.3019766	-2.5287778	C	-0.0152394	0.9069791	-4.7899432
H	0.0045433	1.8117102	-2.1069633	C	-0.0339194	-1.5327332	-4.6548683
C	-0.0014777	0.8043784	-1.6982778	C	-0.0412303	-1.5939603	-6.0535405
C	-0.0163817	-1.7813085	-0.5925655	C	-0.0226513	0.8238332	-6.1843324
C	0.0037804	0.6577736	-0.2854689	H	-0.0052567	1.8844921	-4.3164495
C	-0.0215146	-1.6272820	-1.9628319	H	-0.0512834	-2.5572719	-6.5587785
C	-0.0037804	-0.6577736	0.2854689	H	-0.0183032	1.7347148	-6.7765376
C	0.0163817	1.7813085	0.5925655	H	-0.0413843	-0.4802400	-7.9056535
H	-0.0045433	-1.8117102	2.1069633	Si	0.0383228	2.9040071	3.3555821
H	-0.0218985	-2.7746005	-0.1470260	Si	-0.0383228	-2.9040071	-3.3555821
C	0.0215146	1.6272820	1.9628319	C	-1.5021282	3.9957188	3.3992341
H	0.0218985	2.7746005	0.1470260	H	-1.5160780	4.6107720	4.3065055
C	0.0139342	0.3019766	2.5287778	H	-2.4151049	3.3916619	3.3817195
C	0.0014777	-0.8043784	1.6982778	H	-1.5226146	4.6714625	2.5363223
C	0.0207634	0.2640723	4.0161959	C	1.5963806	3.9709135	3.3833227
C	0.0356578	0.4230545	6.8207886	H	2.4993348	3.3522988	3.3568321
C	0.0152394	-0.9069791	4.7899432	H	1.6294024	4.5859662	4.2900701
C	0.0339194	1.5327332	4.6548683	H	1.6189138	4.6459098	2.5198826
C	0.0412303	1.5939603	6.0535405	C	-1.5963806	-3.9709135	-3.3833227
C	0.0226513	-0.8238332	6.1843324	H	-1.6294024	-4.5859662	-4.2900701
H	0.0052567	-1.8844921	4.3164495	H	-2.4993348	-3.3522988	-3.3568321
H	0.0512834	2.5572719	6.5587785	H	-1.6189138	-4.6459098	-2.5198826
H	0.0183032	-1.7347148	6.7765376	C	1.5021282	-3.9957188	-3.3992341
H	0.0413843	0.4802400	7.9056535	H	2.4151049	-3.3916619	-3.3817195
C	-0.0207634	-0.2640723	-4.0161959	H	1.5160780	-4.6107720	-4.3065055
C	-0.0356578	-0.4230545	-6.8207886	H	1.5226146	-4.6714625	-2.5363223

absolute energy *E* (B3LYP): -1584.40231414 au

[a]Calculated by DFT method [B3LYP/6-31+G(d,p); PCM (CH_2Cl_2)]

Table S8. Coordinates (Å) and Absolute Energy of the Optimized Structure for *trans*-**3b**[a]

atom	x	y	z	atom	x	y	z
C	-0.2446184	-0.5416755	-2.4710920	H	-0.6057576	1.3520772	-4.5771825
H	-0.3358284	1.5981281	-2.2577831	H	-0.5508042	-3.4224313	-6.0243339
C	-0.2276460	0.6397614	-1.7579476	H	-0.8901090	0.7657268	-6.9592286
C	0.0581314	-1.8315493	-0.4133794	H	-0.8698806	-1.6044769	-7.6926710
C	-0.0715294	0.6259118	-0.3454818	P	-0.1779838	-3.1868801	-2.9480687
C	-0.0933529	-1.7847700	-1.7779100	P	0.1779838	3.1868801	2.9480687
C	0.0715294	-0.6259118	0.3454818	O	1.2512640	4.2253009	2.7076971
C	-0.0581314	1.8315493	0.4133794	O	-1.2512640	-4.2253009	-2.7076971
H	0.3358284	-1.5981281	2.2577831	C	-1.4819717	3.9407603	3.0630055
H	0.1676850	-2.7806718	0.1048893	C	-3.9903059	5.1750742	3.2178466
C	0.0933529	1.7847700	1.7779100	C	-2.6318701	3.1724003	3.3068834
H	-0.1676850	2.7806718	-0.1048893	C	-1.5944477	5.3282456	2.8984193
C	0.2446184	0.5416755	2.4710920	C	-2.8481603	5.9428607	2.9763165
C	0.2276460	-0.6397614	1.7579476	C	-3.8813391	3.7891852	3.3833106
C	0.4149327	0.7075551	3.9331449	H	-2.5568819	2.0964166	3.4382574
C	0.7394671	1.3614801	6.6427196	H	-0.7009527	5.9157179	2.7120514
C	0.5905539	-0.3094142	4.8792797	H	-2.9300161	7.0182212	2.8487799
C	0.3969806	2.0532638	4.3604806	H	-4.7677754	3.1909713	3.5718349
C	0.5600779	2.3841517	5.7055429	H	-4.9637957	5.6528230	3.2782097
C	0.7517116	0.0239567	6.2267856	C	1.4819717	-3.9407603	-3.0630055
H	0.6057576	-1.3520772	4.5771825	C	3.9903059	-5.1750742	-3.2178466
H	0.5508042	3.4224313	6.0243339	C	2.6318701	-3.1724003	-3.3068834
H	0.8901090	-0.7657268	6.9592286	C	1.5944477	-5.3282456	-2.8984193
H	0.8698806	1.6044769	7.6926710	C	2.8481603	-5.9428607	-2.9763165
C	-0.4149327	-0.7075551	-3.9331449	C	3.8813391	-3.7891852	-3.3833106
C	-0.7394671	-1.3614801	-6.6427196	H	2.5568819	-2.0964166	-3.4382574
C	-0.5905539	0.3094142	-4.8792797	H	0.7009527	-5.9157179	-2.7120514
C	-0.3969806	-2.0532638	-4.3604806	H	2.9300161	-7.0182212	-2.8487799
C	-0.5600779	-2.3841517	-5.7055429	H	4.7677754	-3.1909713	-3.5718349
C	-0.7517116	-0.0239567	-6.2267856	H	4.9637957	-5.6528230	-3.2782097
absolute energy *E* (B3LYP): -2142.21784297 au							

[a]Calculated by DFT method [B3LYP/6-31+G(d,p); PCM ($CHCl_3$)]

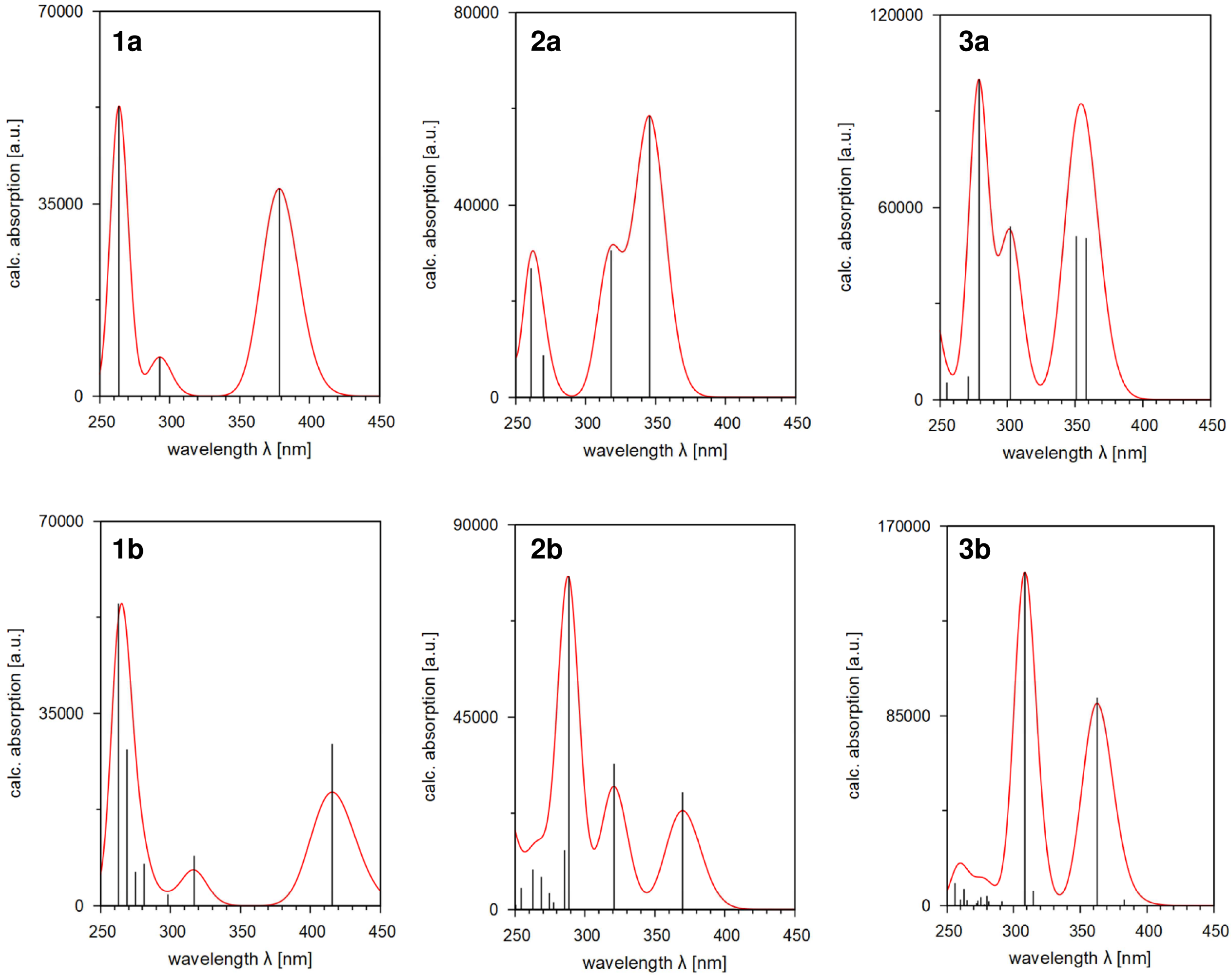

Figure S28. Simulated absorption spectra of **1–3** using TD–DFT method at the B3LYP /6-31+G(d,p) level of theory with PCM (CH_2Cl_2 for **1a**, **2a**, and **3a**; $CHCl_3$ for **1b**, **2b**, and **3b**).

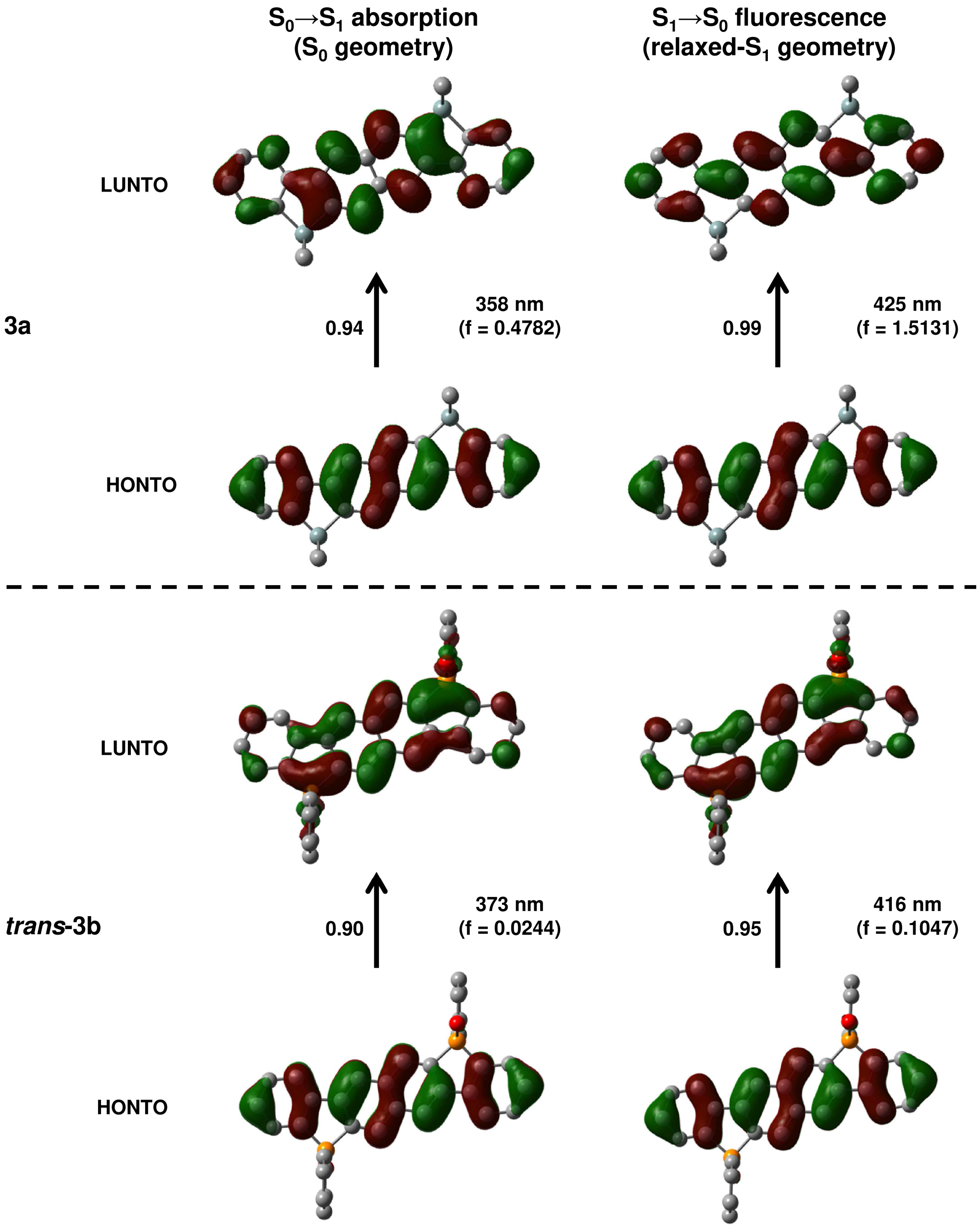

Figure S29. The highest occupied natural transition orbital (HONTO) and the lowest unoccupied natural transition orbital (LUNTO) diagrams corresponding to the S0→S1 (ground-state geometry) and S1 (relaxed excited state)→S0 transitions.

Table S9. The Selected Absorption Peaks of **1–3** Calculated by TD–DFT Method at the B3LYP /6-31+G(d,p) Level of Theory with PCM (CH_2Cl_2 for **1a**, **2a**, and **3a**; $CHCl_3$ for **1b**, **2b**, and **3b**)

	excited state	transition energy (eV)	wavelength (nm)	main transition configuration (CI expansion coefficient)	oscillator strength *f*	Rotatory Strength (10^{-40} erg·esu·cm/gauss)		transition electric dipole moments (a.u.)			transition magnetic dipole moments (a.u.)		
						$R_{velocity}$	R_{length}	x	y	z	x	y	z
**1a**	1	3.2768	378	HOMO → LUMO (0.70091)	0.3730	-0.0000	-0.0000	-0.8888	1.9636	0.0000	0.0000	0.0000	-0.0000
	2	4.2267	293	HOMO−1 → LUMO (0.40621)<br>HOMO → LUMO+2 (0.56532)	0.0000	0.0000	0.0000	0.0000	-0.0000	-0.0000	-0.0000	0.0000	0.8506
	3	4.2317	293	HOMO−2 → LUMO (0.44484)<br>HOMO → LUMO+1 (0.53675)	0.0700	-0.0000	-0.0000	0.2043	-0.7959	0.0000	0.0000	0.0000	-0.0000
	4	4.4945	276	HOMO−4 →LUMO (0.70312)	0.0000	-0.0000	0.0000	0.0000	-0.0000	0.0000	-0.3169	0.3522	0.0000
	5	4.5963	270	HOMO−1 →LUMO (0.53738)<br>HOMO →LUMO+2 (-0.40617)<br>HOMO →LUMO+4 (-0.16666)	0.0000	0.0000	0.0000	-0.0000	0.0000	0.0000	-0.0000	0.0000	-2.0863
**1b**	1	2.9823	416	HOMO → LUMO (0.70163)	0.2038	-0.0000	-0.0000	0.2015	1.0978	1.2422	-0.0000	0.0000	0.0000
	2	3.7544	330	HOMO−7 →LUMO (0.16457)<br>HOMO−5 →LUMO (-0.40328)<br>HOMO −1→LUMO (0.53904)	0.0000	0.0000	-0.0000	0.0000	0.0000	0.0000	0.2773	0.1397	0.0086
	3	3.9123	317	HOMO−3 →LUMO (-0.15984)<br>HOMO−2 →LUMO (0.65410)<br>HOMO →LUMO+3 (-0.18855)	0.0626	0.0000	0.0000	-0.2451	-0.4535	-0.6221	0.0000	0.0000	0.0000
	4	3.9600	313	HOMO−5 →LUMO (0.53409)<br>HOMO−4 →LUMO (-0.16169)<br>HOMO −1→LUMO (0.38893)<br>HOMO →LUMO+1 (-0.14921)	0.0000	0.0000	0.0000	0.0000	-0.0000	-0.0000	-0.7861	0.0830	0.1465
	5	4.0159	309	HOMO−6 →LUMO (0.12488)<br>HOMO−3 →LUMO (0.66504)<br>HOMO−2 →LUMO (0.18708)	0.0009	-0.0000	-0.0000	-0.0356	0.0186	0.0895	-0.0000	0.0000	0.0000

	excited state	transition energy (eV)	wavelength (nm)	main transition configuration (CI expansion coefficient)	oscillator strength f	Rotatory Strength (10^{-40} erg·esu·cm/gauss)		transition electric dipole moments (a.u.)			transition magnetic dipole moments (a.u.)		
						$R_{velocity}$	R_{length}	x	y	z	x	y	z
**2a**	1	3.5841	346	HOMO → LUMO (0.68390)<br>HOMO → LUMO+1 (0.15551)	0.5717	-0.0000	-0.0000	-2.2767	-1.1520	0.0000	-0.0000	0.0000	0.0000
	2	3.8979	318	HOMO−1 → LUMO (0.21871)<br>HOMO → LUMO (-0.15961)<br>HOMO → LUMO+1 (0.64773)	0.2983	0.0000	0.0000	1.5582	0.8341	0.0000	-0.0000	-0.0000	-0.0000
	3	4.3768	283	HOMO−4 → LUMO (-0.19089)<br>HOMO−2 → LUMO (-0.23360)<br>HOMO−2 → LUMO+1 (0.11469)<br>HOMO → LUMO+2 (0.60860)	0.0000	0.0000	-0.0000	-0.0000	0.0000	-0.0000	0.0000	-0.0000	-1.3252
	4	4.5996	270	HOMO−3 → LUMO (0.50466)<br>HOMO−1 → LUMO (-0.30983)<br>HOMO → LUMO+1 (0.10504)<br>HOMO → LUMO+5 (0.33615)	0.0847	0.0000	0.0000	0.7919	-0.3525	-0.0000	0.0000	0.0000	0.0000
	5	4.7302	262	HOMO−4 → LUMO (0.42711)<br>HOMO−2 → LUMO (0.14545)<br>HOMO → LUMO+2 (0.27693)<br>HOMO → LUMO+3 (0.44557)	0.0000	0.0000	0.0000	-0.0000	-0.0000	0.0000	0.0000	-0.0000	-1.0605
**2b**	1	3.3519	370	HOMO → LUMO (0.68979)<br>HOMO → LUMO+1 (-0.12249)	0.2284	0.0407	0.0409	-1.1639	1.1931	0.0519	-0.0001	-0.0002	0.0002
	2	3.8644	321	HOMO−1 → LUMO (-0.28381)<br>HOMO → LUMO (0.11899)<br>HOMO → LUMO+1 (0.61624)	0.2838	0.0132	0.0136	-1.3328	1.1043	0.047	-0.0001	-0.0002	0.0002
	3	4.2715	290	HOMO−5 → LUMO (0.21992)<br>HOMO−2 → LUMO (-0.31081)<br>HOMO−2 → LUMO+1 (-0.10703)<br>HOMO → LUMO+2 (0.54284)<br>HOMO → LUMO+4 (-0.10554)	0.0000	-0.0105	-0.0040	-0.0000	0.0000	-0.0000	0.3360	0.2343	-0.8077
	4	4.2969	289	HOMO−1 → LUMO (0.63812)<br>HOMO → LUMO+1 (0.27200)	0.6488	-0.0042	-0.0043	-2.0349	1.4135	0.1553	-0.0000	-0.0000	0.0001
	5	4.3003	288	HOMO−5 → LUMO (0.14042)<br>HOMO−4 → LUMO (-0.13447)<br>HOMO−2 → LUMO (0.59684)<br>HOMO → LUMO+2 (0.27139)	0.0000	-0.0355	-0.0176	-0.0000	0.0000	-0.0000	0.7785	0.7641	-1.8495

	excited state	transition energy (eV)	wavelength (nm)	main transition configuration (CI expansion coefficient)	oscillator strength f	Rotatory Strength (10^{-40} erg·esu·cm/gauss)		transition electric dipole moments (a.u.)			transition magnetic dipole moments (a.u.)		
						$R_{velocity}$	R_{length}	x	y	z	x	y	z
**3a**	1	3.4609	358	HOMO−1 → LUMO (0.16340)<br>HOMO−1 → LUMO+1 (0.10787)<br>HOMO → LUMO (0.61433)<br>HOMO → LUMO+1 (-0.28244)	0.4782	-0.0000	-0.0000	-0.0154	-0.5762	-2.3039	0.0000	0.0000	-0.0000
	2	3.5345	351	HOMO−1 → LUMO (-0.25790)<br>HOMO → LUMO (0.32410)<br>HOMO → LUMO+1 (0.56593)	0.4833	0.0000	0.0000	-0.0155	-0.6012	-2.2846	-0.0000	0.0000	0.0000
	3	4.1061	302	HOMO−1 → LUMO (0.61307)<br>HOMO → LUMO+1 (0.29966)<br>HOMO → LUMO+4 (-0.11863)	0.5130	0.0000	0.0000	-0.0099	0.1003	-2.2559	-0.0000	0.0000	0.0000
	4	4.2628	291	HOMO−3 → LUMO (0.21430)<br>HOMO−2 → LUMO (0.16496)<br>HOMO−2 → LUMO+1 (-0.15915)<br>HOMO → LUMO+2 (0.58495)<br>HOMO → LUMO+3 (0.18398)	0.0000	0.0000	0.0000	0.0000	-0.0000	0.0000	-1.5510	0.0123	0.0073
	5	4.4153	281	HOMO−2 → LUMO (-0.36801)<br>HOMO → LUMO+3 (0.58344)	0.0000	-0.0000	-0.0000	0.0000	0.0000	0.0000	0.1341	-0.0011	-0.0006
**3b**	1	3.3255	373	HOMO−1 → LUMO (0.19304)<br>HOMO → LUMO (0.33826)<br>HOMO → LUMO+1 (0.57656)	0.0244	-0.0000	-0.0000	-0.0381	-0.0755	-0.5408	-0.0000	0.0000	-0.0000
	2	3.5175	352	HOMO−1 → LUMO+1 (-0.16308)<br>HOMO → LUMO (0.58601)<br>HOMO → LUMO+1 (-0.35086)	0.8881	-0.0000	-0.0000	0.1431	1.2236	2.9645	-0.0000	-0.0000	0.0000
	3	4.0703	305	HOMO−1 → LUMO (0.53657)<br>HOMO−1 → LUMO+1 (-0.38140)<br>HOMO → LUMO (-0.15202)<br>HOMO → LUMO+1 (-0.14953)	0.0639	0.0000	0.0000	0.0520	-0.1322	0.7876	-0.0000	0.0000	-0.0000
	4	4.1226	301	HOMO−3 → LUMO+1 (-0.11427)<br>HOMO−2 → LUMO (0.24146)<br>HOMO−2 → LUMO+1 (0.16751)<br>HOMO → LUMO+2 (0.59547)	0.0000	0.0000	0.0000	0.0000	-0.0000	-0.0000	-1.8354	-0.1216	0.2683
	5	4.1558	298	HOMO−1 → LUMO (0.39374)<br>HOMO−1 → LUMO+1 (0.54783)<br>HOMO → LUMO (0.11543)<br>HOMO → LUMO+1 (-0.11499)	1.4255	-0.0000	-0.0000	-0.1341	1.5928	3.3833	-0.0000	-0.0000	0.0000
